# Detailed Mitochondrial Phenotyping by High Resolution Metabolomics

**DOI:** 10.1371/journal.pone.0033020

**Published:** 2012-03-06

**Authors:** James R. Roede, Youngja Park, Shuzhao Li, Frederick H. Strobel, Dean P. Jones

**Affiliations:** 1 Division of Pulmonary, Allergy and Critical Care Medicine, Department of Medicine, Emory University, Atlanta, Georgia, United States of America; 2 Emory Vaccine Center, Yerkes National Primate Center, Emory University, Atlanta, Georgia, United States of America; 3 Mass Spectrometry Center, Emory University, Atlanta, Georgia, United States of America; University of Colorado Denver, United States of America

## Abstract

Mitochondrial phenotype is complex and difficult to define at the level of individual cell types. Newer metabolic profiling methods provide information on dozens of metabolic pathways from a relatively small sample. This pilot study used “top-down” metabolic profiling to determine the spectrum of metabolites present in liver mitochondria. High resolution mass spectral analyses and multivariate statistical tests provided global metabolic information about mitochondria and showed that liver mitochondria possess a significant phenotype based on gender and genotype. The data also show that mitochondria contain a large number of unidentified chemicals.

## Introduction

Mitochondria are the main source of energy in most eukaryotic cells. They play a vital role in ATP production via oxidative phosphorylation and the tricarboxylic acid cycle (TCA cycle). While energy production is a major process performed by the mitochondrion, many other important metabolic pathways are present. These pathways include fatty acid and amino acid oxidation, apoptosis, and biosynthesis of ketone bodies, pyrimidines, steroids, heme and urea [1]. Due to the essential role that they play in cellular metabolism, dysfunction of this organelle can have dire consequences.

Mitochondrial dysfunction represents a generic disease mechanism, i.e., if mitochondria do not function properly, disease will result [2]. Mitochondrial dysfunction is a primary cause of many inborn errors of metabolism. For example, Kearns-Sayre syndrome results from large deletions of the mitochondrial genome and maple syrup urine disease is caused by a deficiency in branched chain amino acid metabolism [3], [4]. Mitochondrial dysfunction can also occur as a result of a toxic insult or disease pathogenesis. For example, the potent antineoplastic agent, doxorubicin, can cause cardiotoxicity via oxidative stress and redox cycling in cardiomyocyte mitochondria [5]. Additionally, Complex I inhibition and subsequent neurotoxicity has been implicated in pesticide-mediated Parkinson's disease [6]. Lastly, the mitochondrial protein, thioredoxin-2 (Trx2), is intimately involved in the maintenance of cell viability and mitochondrial redox potential. For example, Trx2 is a key defense mechanism against oxidant-induced apoptosis and regulator of mitochondrial permeability transition [7], [8]. It is unknown, however, how alterations in abundance of Trx2 will affect mitochondrial and whole cell metabolism.

Environmental influences, including diet, chemical exposures and lifestyle, vary widely amongst individuals. Additionally, environmental exposures play a major role in chronic disease [9]. Because of this, Wild [9] emphasized the need to complement the human genome project with an effort to characterize the spectrum of exposures that an individual experiences, the “exposome”, and how these exposures impact disease risk. Metabolic profiling of human plasma can give valuable information regarding all environmental influences, in addition to genetics, epigenetics and proteomics, affecting health outcome and disease [10]. Common approaches employed to profile metabolites are nuclear magnetic resonance (NMR) and mass spectrometry. Recently, the development of high resolution, high mass accuracy mass spectrometers, such as Fourier transform ion cyclotron resonance (FTICR) and Orbitrap mass spectrometry, have allowed for accurate mass determination and improved throughput due to a decreased need for lengthy separation techniques and ion fragmentation [10], [11].

Much is known about the mitochondrial proteome [1]; however, knowledge about the spectrum of metabolites in the mitochondria has largely been obtained from studies of specific pathways. Targeted studies have examined changes in mitochondrial metabolism by analyzing peripheral fluids like urine and plasma or whole cells in culture [12], [13], [14], [15], [16], [17], [18], [19]. In contrast, this “top-down” method of high performance metabolic profiling [10], [20] examines the array of metabolites present in isolated mitochondria, identified in terms of high-resolution mass-to-charge ratio (*m/z*) and chromatographic retention time. Because the high-resolution *m/z* has sufficient mass accuracy to predict elemental composition, and >90% of the most common intermediary metabolites (e.g. approximately 2000 present in human metabolomics databases) are non-redundant in elemental composition, one can match *m/z* and metabolic pathways, and verify identities by MS/MS and retention time relative to authentic standards [10], [20].

In the present study, mitochondrial isolations from wild-type and mice over-expressing the mitochondrial protein, thioredoxin-2, were extracted using acetonitrile and analyzed via high performance metabolic profiling involving liquid chromatography and Fourier Transform ion cyclotron resonance mass spectrometry (LC-FTMS). Employing a column switching technique that utilizes both anion exchange and reverse phase chromatography, coupled to FTMS, more than 2,100 mitochondrial metabolites were identified. Statistical analysis using false discovery rates revealed more than 200 *m/z* that distinguish male and female mitochondrial fractions. Partial least squares discriminant analysis indicated that over 100 *m/z* contributed to differences between mitochondria from wild-type (WT) and thioredoxin-2 transgenic (TG). The results suggest that implementation of this procedure could provide a useful hypothesis generating approach to identify mitochondrial metabolic pathways contributing to toxicity or disease pathogenesis. The large number of *m/z* not represented by chemicals in the metabolomics databases also raises the possibility that unidentified metabolic processes exist in the mitochondria.

## Materials and Methods

### Animals and liver harvest

All procedures involving animals were approved by the Institutional Animal Care and Use Committee of Emory University and were performed in accordance with published National Institutes of Health guidelines. A total of 40 animals (wild type (n = 20); Trx2Tg (n = 20)) were used in this study and were separated into groups (n = 5) based on sex (male; female), age (<6 mo; >12 mo) and genotype (WT; TG). Mice were anesthetized with CO_2_ and euthanized via cervical dislocation. The liver was excised immediately and placed into homogenization buffer (22 mM mannitol, 70 mM sucrose, 2 mM Hepes (pH 7.4), 1 mM EGTA). Mitochondria were isolated by differential centrifugation using a method adapted from Savage et al. [21]

### Sample preparation and extraction

After isolation of mitochondria a small aliquot was used in the determination of the protein content via BCA assay. To ensure that equivalent amounts of mitochondria would be analyzed, a sample corresponding to 250 µg of mitochondrial protein was used and diluted to a final volume of 50 µl using incubation buffer (250 mM sucrose, 10 mM MOPS, 3 mM potassium phosphate, 5 mM succinate and 5 mM malate (pH 7.4)). A standard mix consisting of 14 stable isotopic chemicals that represent a broad range of chemical properties was added to each sample prior to extraction and analysis as per Soltow et al. [20]. Metabolites were extracted by adding 100 µl of acetonitrile to each 50 µl sample and vortexed to mix. The precipitated protein was pelleted via centrifugation and the supernatant was used for metabolic profiling.

### LC-FTMS metabolic profiling

Details of the high performance metabolic profiling method have been published [10], [20]. Briefly, mitochondrial extracts in acetonitrile were separated on either a C_18_-reverse phase column or strong anion exchange column [20]. The eluate from the HPLC separation was connected to a Thermo LTQ-FT mass spectrometer (Thermo Fisher Scientific, San Jose, CA). Analyses were performed using the MS^1^ mode scanning from a *m/z* range of 85–850 in the FT detector at a resolution of 50,000 with the wide range scan mode and 3 million ions per scan. The maximum ion injection time was 500 ms. Each sample was run in duplicate with a 10 µL injection volume on each column, taking approximately one hour to analyze each sample in quadruplicate.

### Data collection, processing and analysis

Data were collected as described by Johnson et al. Data were processed using the R package apLCMS [22]. To determine if an *m/z* feature was a mitochondrial metabolite or a buffer component, buffer only samples were analyzed and a sample-to-buffer ratio (s/b) (ratio of sample *m/z* to corresponding *m/z* in buffer sample) was calculated. A specific *m/z* was categorized as a mitochondrial metabolite if it was found only in the mitochondrial sample or if s/b ≥4. False discovery rate (FDR) and orthogonal partial least squares – discriminant (OPLS-DA) analyses were performed using 1,485 *m/z* from anion exchange column and 1,111 *m/z* from C18 column. FDR [23], [24] was performed using MATLAB to identify *m/z* to discriminate between two groups. For OPLS-DA, variation not correlated to classification was removed with orthogonal signal correction (OSC) using Pirouette software (Infometrix, Bothell, WA) after centering the mean value for each frequency [25].

### Pathway analysis

The pathway mapping was performed by an in-house program. Our program considers all common derivatives as given by Brown et al. [26] when comparing *m/z* values to the theoretical molecular weights of metabolites. The stringency of such comparison was guided by a calibration curve on our FTICR spectrometer, which agrees with the inverse correlation between FTMS resolution and *m/z* [27]. This calibration curve indicates accuracy of 10 ppm at about 300 m/z, while less accuracy at higher *m/z*. The data for KEGG (Kyoto Encyclopedia of Genes and Genomes) pathways and compounds were retrieved from the KEGG FTP site (retrieved on April 26, 2011). The KEGG human metabolic pathways contain 1486 metabolites. The visualization of the mapped compounds on KEGG metabolic map is performed via the “Color Pathway”tool in KEGG Mapper (http://www.genome.jp/kegg/tool/color_pathway.html).

## Results

### Study design and metabolite distribution

**Figure 1:**
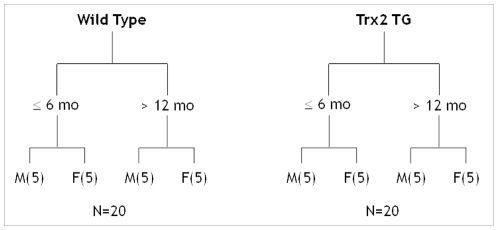
A breakdown of the animals used for mitochondrial isolation and metabolic profiling studies. A total of 40 animals were used, 20 wild type and 20 thiroredoxin-2 transgenic (Trx2TG). These groups were further broken down into subgroups of 5 based on age and sex.

To study the spectrum of metabolites localized to mitochondria and to analyze any phenotypic differences attributed to age, sex or genotype, we isolated mitochondria from WT and TG mice (Figure 1). Mitochondrial isolations from male, female and mice aged less than 6 months or greater than 12 months were analyzed. After isolation, metabolites were extracted using acetonitrile (ACN) and analyzed via LC-FTMS. After processing with apLCMS, anion exchange chromatography (AE) resolved 2872 *m/z* and reverse phase (C18) chromatography resolved 2754 *m/z*.

**Figure 2:**
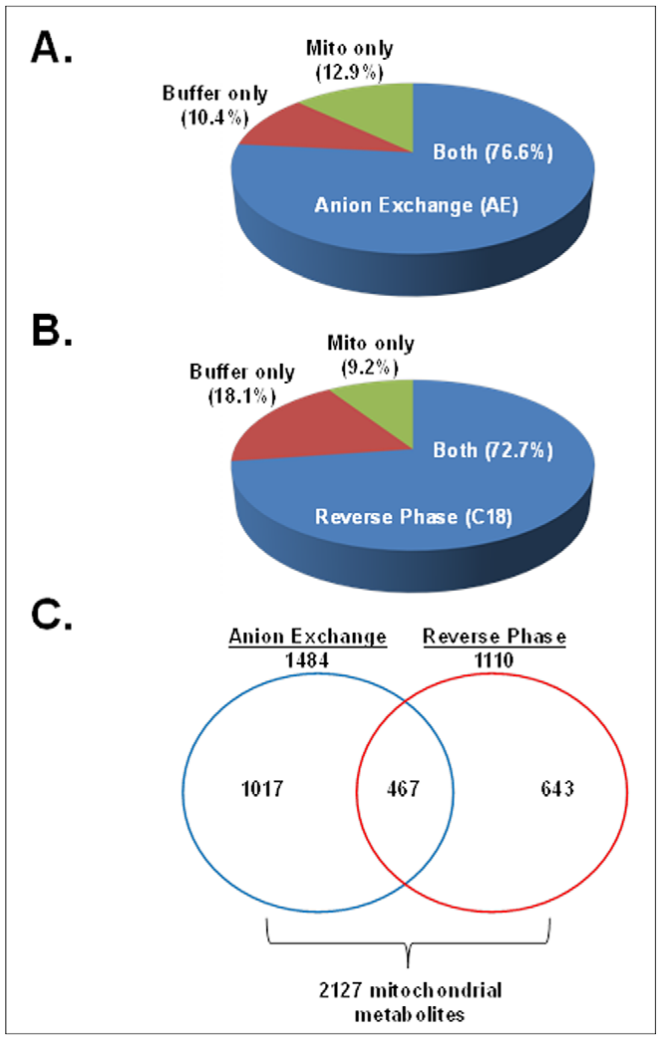
Distribution of metabolites resolved by anion exchange (A) and reverse phase (B) chromatography. To determine if a metabolite was present in the mitochondrial isolation and not a buffer contaminant, a ratio of ion intensity (sample ion intensity/buffer ion intensity) was calculated for each metabolite. A metabolite was determined to be “mitochondrial” if this ratio (s/b) was greater than or equal to 4. Additionally, employment of two chromatographic techniques resulted in the detection of 2127 mitochondrial metabolites (C).

Samples of the mitochondrial isolation buffer were also analyzed to discern what *m/z* corresponded to actual mitochondrial metabolites and buffer components. A ratio of sample ion intensity to buffer ion intensity for each *m/z* was calculated (Figure 2). Metabolites with a ratio (s/b) ≥4 were considered to be mitochondrial and not buffer contaminants. This additional processing resulted in 1484 and 1110 *m/z* for AE and C18 respectively. Greater than 70% of the *m/z* had s/b <4 (Figure 2A&B); therefore, it is probable that the total number of mitochondrial metabolites assigned in this study is an underestimation. Additionally, the data from the AE and C18 columns were compared to determine what mitochondrial *m/z* were common for both chromatographic techniques (Figure 2C). This analysis showed that 467 *m/z* were common for both AE and C18, while 1017 and 643 were unique for AE and C18 respectively. Together, these results show that this high performance metabolic profiling technique resolved 2127 unique *m/z*.

### Results of metabolic pathway analysis and feature annotation

**Figure 3:**
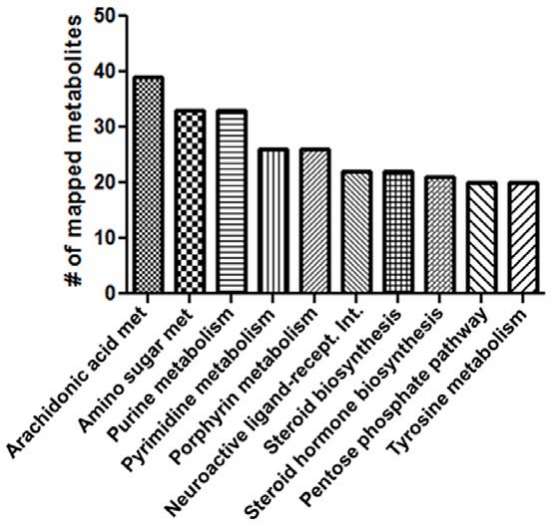
Most metabolic pathways are represented in metabolites isolated from liver mitochondria. Ten pathways containing the most matches are plotted.

Mitochondria are involved in many metabolic pathways, such as oxidative phosphorylation, TCA cycle, fatty acid metabolism and amino acid oxidation. To test whether the mitochondrial *m/z* mapped to metabolic pathways, we used KEGG pathway analysis to investigate. The human KEGG pathways contain 1486 specific metabolites. Analyses showed that 745 *m/z* from AE and C18 matched known KEGG metabolites. Of these, at least one metabolite mapped to 136 of the 154 possible KEGG pathways. It should be noted, however, that, of these 745 *m/z*, many can be considered as ‘benchmark’ metabolites and can be present in multiple pathways. The 10 pathways that received the most matches are shown in Figure 3 and included arachidonic acid, amino sugar, nucleotide and steroid metabolism.

To further annotate the data, *m/z* were analyzed for matches within 10 ppm with the Madison Metabolomics Consortium Database (MMCD) and Metlin databases. These databases contain information regarding the identification of specific molecules according to their *m/z* and common ionic forms, e.g. proton ([M+H]), sodium ([M+Na]), and acetonitrile ([M+ACN*+*H]) adducts. Identities of some *m/z* matches to metabolomics databases were confirmed using internal standards, including amino acids and a relatively small number of nutrients, dietary compounds, intermediary metabolites and environmental compounds. For example, the identity of glucose, methionine, glutamate and tyrosine were all confirmed by *m/z* and retention time matching to stable isotopic controls. Because confirmation of identity of the total number of metabolites was not feasible, we used bioinformatic methods to test for differences according to sex, genotype and age.

### False discovery rate analysis reveals a sex phenotype

False discovery rate analysis (FDR) is an extension of a Bonferroni-type, multiple comparison procedure that is used to determine what *m/z* contribute significantly to discriminate groups. Therefore, FDR was employed to determine if mitochondrial metabolic profiles were significantly different between males vs. females, WT vs. TG, and young vs. old. Because of the discovery/exploratory nature of these studies, the q-value was set at 0.1. FDR did not show significance due to age (<6 mo. vs. ≥12 mo.) or genotype (WT vs. TG). However, a significant difference was observed due to sex. This analysis showed that 246 *m/z* from AE and 211 *m/z* from C18 distinguished male mitochondria from female mitochondria (Table S1A&B). Briefly, putative identification of these *m/z* included amino acids, dipeptides, tripeptides, nucleotides and a number of other known and unknown metabolites.

**Figure 4:**
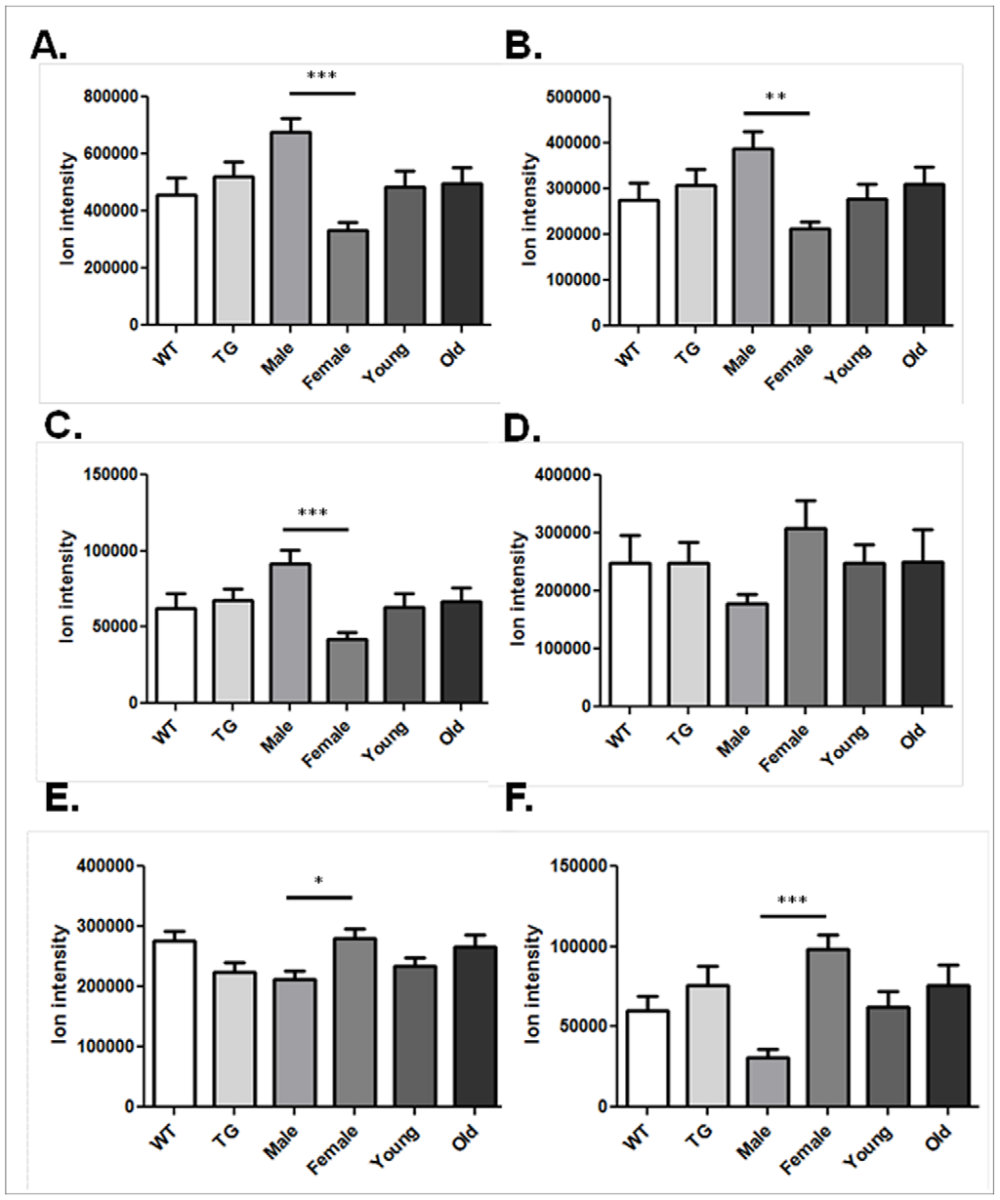
Mitochondrial metabolites from AE that were found to discriminate male from female mitochondria using false discovery rate analysis (q = 0.1). Metabolites with greater ion intensity in male mitochondria include (A) leucine/isoleucine, (B) glutamate and (C) methionine. Metabolites with greater ion intensity in female mitochondria include (D) adenosine, (E) amino octadecanoic acid and (F) unknown metabolite (*m/z* 537.789). Data was analyzed using one-way ANOVA and Tukey's post hoc test (* p<0.05, ** p<0.01, *** p<0.001).

Of the 246 AE *m/z* found to distinguish male from female, 197 *m/z* had greater ion intensity in male mitochondria while 49 had greater intensity in females. A sample of 3 *m/z* with greater ion intensity in males (Figure 4A–C) includes amino acids, leucine/isoleucine, glutamate, and methionine. Sample metabolites with greater ion intensity in females (Figure 4D–E) include adenosine, amino octadecanoic acid and an unknown metabolite (*m/z*: 537.789).

**Figure 5:**
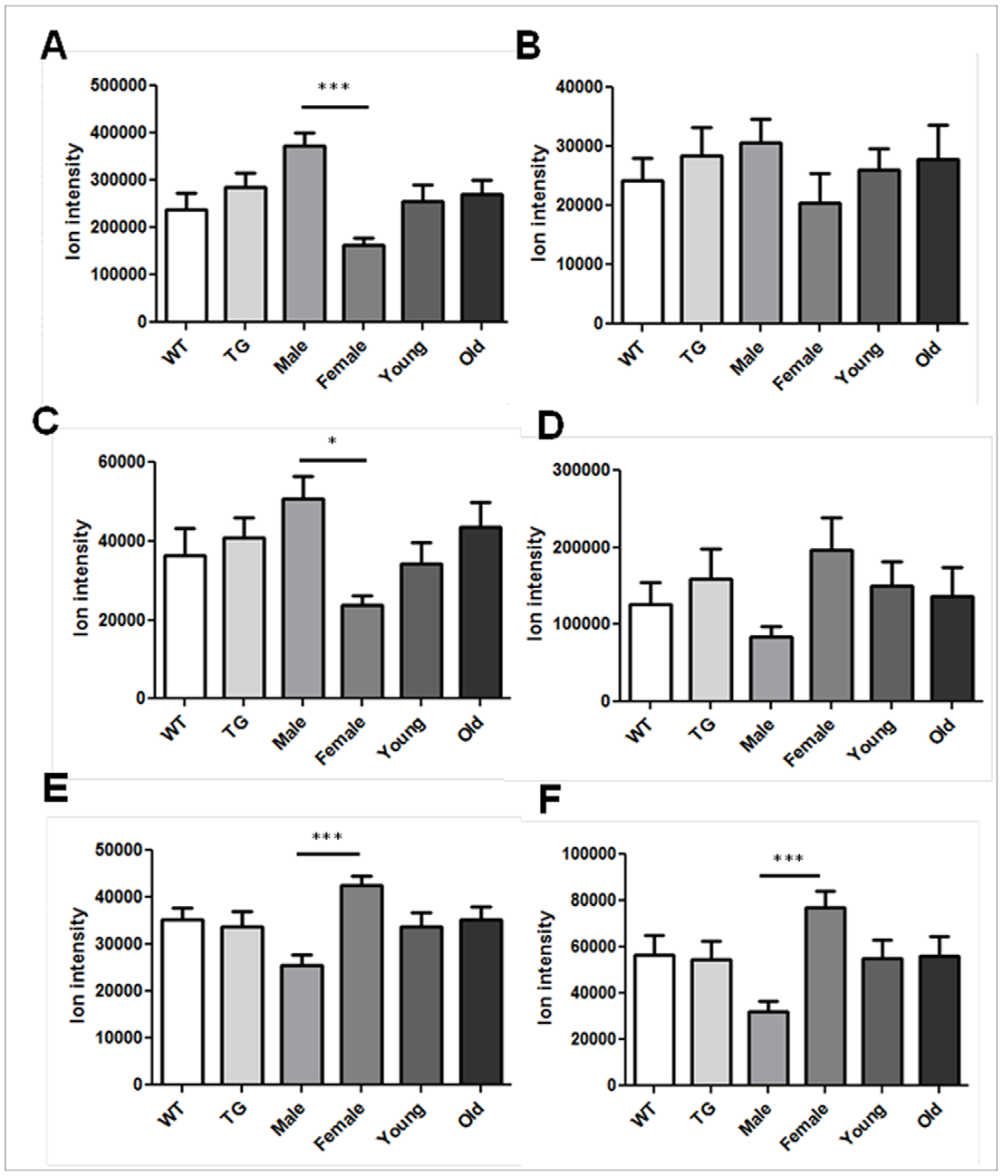
Mitochondrial metabolites from C18 that were found to discriminate male from female mitochondria using false discovery rate analysis (q = 0.1). Metabolites with greater ion intensity in male mitochondria include (A) leucine/isoleucine, (B) glutamate and (C) methionine. Metabolites with greater ion intensity in female mitochondria include (D) adenosine, (E) sphinganine and (F) unknown metabolite (*m/z* 442.759). Data was analyzed using one-way ANOVA and Tukey's post hoc test (* p<0.05, *** p<0.001).

FDR also determined that 211 *m/z*, resolved using C18, distinguished male from female mitochondria. 178 *m/z* had greater ion intensity in male mitochondria and 33 had greater ion intensity in female mitochondria. Figure 5 shows a sample of 6 *m/z* that had greater ion intensity in either males or females. Metabolites with greater ion intensity in males included leucine/isoleucine, glutamate, and methionine (Figure 5A–C). Additionally, similar to that of AE, adenosine had higher ion intensity in females along with sphinganine and an unknown metabolite (*m/z*; 442.759) (Figure 5D–F).

### Partial least squares discriminant analysis shows significant difference between WT and TG

**Figure 6:**
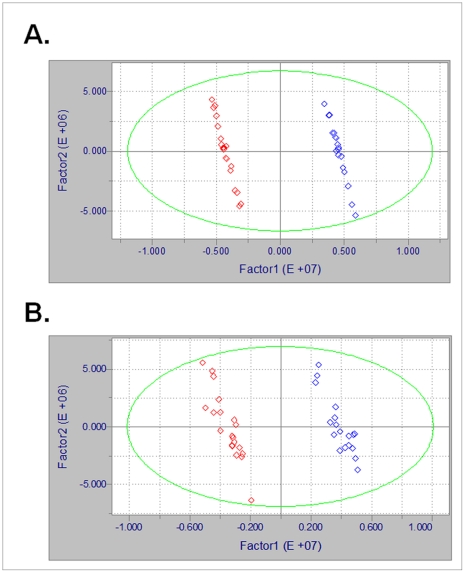
OPLS-DA score plot results comparing WT vs. TG mitochondrial metabolites from (A) AE and (B) C18. WT (red) and TG (blue).

We used partial least squares discriminant analysis with orthogonal signal correction (OPLS-DA) to further evaluate whether metabolic differences between WT and TG mice could be detected. Score plots of the OPLS-DA results show that data for both AE (Figure 6A) and C18 (Figure 6B) clustered into two distinct groups. OPLS-DA of data from AE showed that signals from 108 *m/z* contributed to separation while C18 analysis showed contribution of 136 *m/z*. Of these 244 *m/z*, 38 were common to both chromatographic techniques, resulting in 206 distinguishing *m/z*. Putative identification of these *m/z* included many methylated metabolites and other molecules involved in methyl group donation. These compounds included methylated nucleotides, folate and choline (Table S2A&B).

## Discussion

Metabolic profiling involves the measurement and characterization of metabolites and is typically applied to samples acquired though minimally invasive methods, such as plasma and urine. However, this current project highlights information that can be attained by profiling isolated mitochondria. Our method of high performance metabolic profiling commonly used on plasma samples was applied to isolated liver mitochondria. Use of a simple organic extraction and established LC-FTMS protocol with data extraction by apLCMS resulted in the detection of more than 2,100 mitochondrial metabolites. It is important to note that greater than 70% of the *m/z* detected were present in both the mitochondrial samples and buffer controls. Thus, it is likely that this number of mitochondrial metabolites is an underestimation due to the natural occurrence of the buffer components in mammalian cells, such as succinate, malate and various 6-carbon carbohydrates. It is important to additionally note that the mitochondrial preparation is primarily mitochondria but also contains lysosomes and peroxisomes. Some of the metabolites may be unique to these organelles, but fractionation studies were not pursued because of the likelihood of artifactual metabolite loss due to additional manipulations required for separation.

KEGG is a widely used compendium of biological pathways that consists of genomic, chemical, and network information with cross references to outside databases [28], [29]. Using KEGG pathway analysis, we found that at least one mitochondrial metabolite mapped to over 88% (136/154) of the KEGG pathways. Importantly, the pathways that had the greatest number of matches were also known to involve the mitochondria. For example, 22 and 21 metabolites mapped to steroid biosynthesis and steroid hormone biosynthesis, respectively. This is important because the rate limiting step in steroid biosynthesis is transport of cholesterol into the mitochondria for the conversion of cholesterol to pregnenolone [30]. Also, porphyrin metabolism came up with the fourth most hits and four of the eight steps involved in heme biosynthesis take place in the mitochondrion [31]. Of general importance, this pathway data demonstrates that mitochondria contain metabolites from the majority of cellular metabolic pathways.

Unsupervised statistical analyses comparing sex, age or genotype uncovered only a sex phenotype. FDR analysis provided 311 *m/z* that distinguished the two sexes. In particular, male mitochondria had greater ion intensities for amino acids when compared to females. Lamont and colleagues reported gender differences in amino acid metabolism in mitochondria from skeletal muscle. They found that, during exercise, females relied more on fat as an energy source where males utilized amino acid oxidation [32], [33]. Additionally, estrogen enhances transcription of genes encoded by mitochondrial DNA, resulting in an increase in the functional capacity of female mitochondria and may potentially protect young females from β-amyloid toxicity [34], [35], [36]. Taken together, the data show that use of high performance metabolic profiling is suitable for discovery of phenotypic differences in mitochondrial metabolism.

While a significant difference was not detected when WT and TG mitochondria were compared using FDR, the supervised test, OPLS-DA, was successful in identifying 206 *m/z* that distinguish the mitochondria. Putative identification of these *m/z*, such as tetrahydrofolate 10-formyldihydrofolate and cytidine monophosphate, revealed a number of molecules involved in choline metabolism and purine and pyrimidine metabolism. These data indicate a possible phenotype involving methylation pathways. Supporting this phenotype, unpublished proteomic data from our laboratory (Y-M Go, unpublished) comparing protein expression in WT versus TG mice showed differences in expression of sarcosine dehydrogenase and betaine-homocysteine methyltransferase. Sarcosine dehydrogenase converts monomethyl-glycine to glycine and betaine-homocysteine methyltransferase transfers a methyl group from trimethyl-glycine to homocysteine, producing methionine and dimethyl-glycine. Both of these enzymes are involved in the catabolism of choline and glycine [37]. Alterations in metabolic profiles have also been observed in other studies involving transgenic animals. For example, Manna and colleagues showed that PPAR-α null mice, when exposed to ethanol, had an increased amount of indole-3-lactone in the urine [38]. Also, a metabolomic comparison of three different transgenic models of Huntington's disease (HD) revealed that all three models shared four common metabolites that were indicative of the HD phenotype [39].

### Conclusion

The data presented in this manuscript demonstrate that high performance metabolic profiling can be applied to isolated mitochondria to discover metabolic phenotypes that might be difficult to discern by targeted approaches. The KEGG pathway analyses show that mitochondria contain metabolites from a majority of cellular metabolic pathways. Statistical comparisons of the samples based on sex, age and genotype show that both sex and the over-expression of the mitochondrial protein, Trx2, result in a distinct phenotype. Additionally, the large number of unidentified *m/z* in the mitochondrial preparations implicate several different possibilities, such as the presence of metabolic pathways yet to be described or an extensive accumulation of environmental chemicals, some of which could be retained because of their lipophilic, cationic nature and no effective elimination pathways. This would suggest a need to further examine detoxification functions of mitochondrial cytochrome P450 enzymes. These pathways would be of particular importance under pathologic conditions, such as alcohol abuse, where GSH metabolism is impaired. With this in mind, the results suggest that this approach could be valuable in hypothesis development and discovery of new mechanisms and biomarkers involved in mitochondrial dysfunction, such as profiling human fibroblasts from infants with unidentified disorders or as a diagnostic tool for inborn errors of metabolism.

## Supporting Information

Table S1 Annotated mitochondrial metabolites from AE (A) and C18 (B) found to be significant between male and female via FDR. Features found to be significant were searched against the metabolomics databases (MMCD and Metlin) to assign reasonable matches. Metabolites that matched to known human drugs and drug metabolites were excluded.
(DOC)

Table S2 Annotated mitochondrial metabolites from AE (A) and C18 (B) found to be significant between WT and TG via OPLS-DA. Features found to be significant were searched against the metabolomics databases (MMCD and Metlin) to assign reasonable matches. Metabolites that matched to known human drugs and drug metabolites were excluded.
(DOC)
