# Supplementary material for: Detailed Mitochondrial Phenotyping by High Resolution Metabolomics
Source: PLoS One. 2012 Mar 6;7(3):e33020. doi: 10.1371/journal.pone.0033020 (PMC3295783; doi:10.1371/journal.pone.0033020)
Supplement: Table S1 — Annotated mitochondrial metabolites from AE (A) and C18 (B) found to be significant between male and female via FDR. Features found to be significant were searched against the metabolomics databases (MMCD and Metlin) to assign reasonable matches. Metabolites that matched to known human drugs and drug metabolites were excluded. (DOC) [file pone.0033020.s001.doc]

**Table S1.**

A.

| **p-value** | **m/z** | **RT** | **Identity** | **formula** | **adduct** |
| --- | --- | --- | --- | --- | --- |
| 4.2513E-07 | 132.1015 | 73.7 | Leucine/Isoleucine | C6H13NO2 | [M+H] |
| 4.2513E-07 | 132.1015 | 73.7 | 2,3-Butanediol | C4H10O2 | [M+ACN+H] |
| 1.7772E-10 | 147.0762 | 75.7 | Glutamine | C5H10N2O3 | [M+H] |
| 1.7772E-10 | 147.0762 | 75.7 | Serine | C3H7NO3 | [M+ACN+H] |
| 0.00017603 | 147.1131 | 62.0 | CARBACHOL | C6H14N2O2 | [M+H] |
| 0.00016803 | 148.0595 | 105.7 | Glutamate | C5H9NO4 | [M+H] |
| 7.4056E-06 | 150.0580 | 75.2 | Methionine | C5H11NO2S | [M+H] |
| 0.0111493 | 160.0963 | 232.8 | N-isovalerylglycine; 3-dehydrocarnitine; 2-methylbutyrylglycine | C7H13NO3 | [M+H] |
| 0.0111493 | 160.0963 | 232.8 | DL-a-Hydroxyisovalerate | C5H10O3 | [M+ACN+H] |
| 2.5136E-06 | 166.0859 | 74.8 | Phenylalanine | C9H11NO2 | [M+H] |
| 0.00071902 | 168.0687 | 75.6 |  |  |  |
| 2.2165E-05 | 182.0807 | 75.3 | Tyrosine | C9H11NO3 | [M+H] |
| 0.00082546 | 184.0635 | 78.9 |  |  |  |
| 9.4967E-05 | 189.1238 | 67.3 | 2,5-undecadienal | C8H9NO3 | [M+Na] |
| 9.4967E-05 | 189.1238 | 67.3 | Aceyllysine; Dipeptide: Gly-Leu/Ile; Ala-Val | C8H16N2O3 | [M+H] |
| 0.00035472 | 192.0684 | 235.7 |  |  |  |
| 0.00039409 | 196.1000 | 75.7 |  |  |  |
| 0.00100707 | 198.0793 | 77.7 |  |  |  |
| 0.00108955 | 203.1395 | 66.5 | 3,6,8-dodecatrien-1-ol | C12H20O | [M+Na] |
| 0.00108955 | 203.1395 | 66.5 | Dipeptide: Ala-Leu/Ile | C9H18N2O3 | [M+H] |
| 0.00108955 | 203.1395 | 66.5 | L-Carnitine | C7H15NO3 | [M+ACN+H] |
| 0.01054341 | 205.0966 | 75.9 | Tryptophan | C11H12N2O2 | [M+H] |
| 0.01054341 | 205.0966 | 75.9 | p-acetaminobenzaldehyde | C9H9NO2 | [M+ACN+H] |
| 2.7223E-05 | 210.1153 | 75.1 |  |  |  |
| 0.00013133 | 211.1192 | 75.1 | N,N-dimethyltryptamine | C12H16N | [M+Na] |
| 0.00013133 | 211.1192 | 75.1 | 1-methylhistidine | C7H11N3O2 | [M+ACN+H] |
| 3.6152E-05 | 215.1395 | 73.0 | 2,4,7-tridecatrienal | C13H20O | [M+Na] |
| 3.6152E-05 | 215.1395 | 73.0 | Dipeptide: Val-Pro | C10H18N2O3 | [M+H] |
| 3.6152E-05 | 215.1395 | 73.0 | Isovalerylalanine | C8H15NO3 | [M+ACN+H] |
| 0.00406376 | 219.1345 | 68.0 | Aleprylic acid; 2E,4E-dodecadienoic acid | C12H20O2 | [M+Na] |
| 0.00406376 | 219.1345 | 68.0 | Dipeptide: Val-Thr; Leu/Ile-Ser | C9H18N2O2 | [M+H] |
| 0.01308492 | 219.1517 | 65.9 |  |  |  |
| 0.00177131 | 228.0722 | 76.5 |  |  |  |
| 1.4753E-05 | 229.1552 | 71.6 | Dipeptide: Pro-Leu/Ile | C11H20N2O3 | [M+H] |
| 1.4753E-05 | 229.1552 | 71.6 | 8-amino-7-oxononanoate | C9H17NO3 | [M+ACN+H] |
| 0.00087413 | 231.1709 | 66.3 | 5,8-tetradecadienal | C14H24O | [M+Na] |
| 0.00087413 | 231.1709 | 66.3 | Dipeptide: Leu/Ile-Val | C11H22N2O3 | [M+H] |
| 0.00354209 | 233.1502 | 66.8 | Tridicynoic acid | C13H22O2 | [M+Na] |
| 0.00354209 | 233.1502 | 66.8 | Dipeptide: Leu/Ile-Thr | C10H20N2O4 | [M+H] |
| 0.00015007 | 237.1240 | 66.4 | Dipeptide: Phe-Ala | C12H16N2O3 | [M+H] |
| 0.00015007 | 237.1240 | 66.4 | 2-phenyl-1,3-propanediyl monocarbamate | C10H13NO3 | [M+ACN+H] |
| 0.00231705 | 239.1052 | 159.9 | 2-naphthalenepropanol, 6-hydroxy-a-methyl; 10E,12E-tetradecadien-4,6-diynoic acid | C14H16O2 | [M+Na] |
| 0.00231705 | 239.1052 | 159.9 | 4\'-METHOXYCHALCONE | C16H14O2 | [M+H] |
| 0.00050642 | 240.1090 | 75.6 | 2-benzimidazolinone, 1-(4-piperidyl)- | C12H15N3O | [M+Na] |
| 0.00050642 | 240.1090 | 75.6 | Dihydrobiopterin; 6-lactoyltetrahydrobiopterin; 4a-carbinolamine tetrahydrobiopterin | C9H13N5O3 | [M+H] |
| 0.00017646 | 244.0999 | 76.1 |  |  |  |
| 0.01391492 | 244.1379 | 164.8 |  |  |  |
| 0.00033287 | 245.1866 | 65.9 | Polyprenol; nerolidol; 2,4-pentadecadienal | C15H26O | [M+Na] |
| 0.00033287 | 245.1866 | 65.9 | Dipeptide: Leu/Ile-Leu/Ile | C12H24N2O3 | [M+H] |
| 0.00905951 | 246.1453 | 69.9 | a-[1-(diethylamino)ethyl]-p-hydroxy-benzyl alcohol | C13H21NO2 | [M+Na] |
| 0.00905951 | 246.1453 | 69.9 | Dipeptide: Val-Gln; Asn-Leu/Ile Tripeptide: Gly-Gly-Leu/Ile; Ala-Gly-Val | C10H19N3O4 | [M+H] |
| 0.00905951 | 246.1453 | 69.9 | Dipeptide: Val-Ser | C8H16N2O4 | [M+ACN+H] |
| 0.00256319 | 246.1823 | 61.1 | Dipeptide: Lys-Val | C11H23N3O3 | [M+H] |
| 2.5301E-05 | 258.1085 | 106.7 | 5-methylcytidine; glycerophosphocholine | C10H15N3O5; C8H20NO6P | [M+H] |
| 0.00013468 | 260.0951 | 76.8 |  |  |  |
| 0.00749117 | 260.1610 | 67.8 | Dipeptide: Gln-Ile/Leu Tripeptide: Ala-Gly-Leu/Ile; Ala-Ala-Val | C11H21N3O4 | [M+H] |
| 0.00749117 | 260.1610 | 67.8 | Dipeptide: Ser-Ile/Leu; Thr-Val | C9H18N2O4 | [M+ACN+H] |
| 0.00175228 | 260.1979 | 58.6 | Dipeptide: Leu/Ile-Lys | C12H25N3O3 | [M+H] |
| 0.0003283 | 261.1448 | 68.3 | (9R, 13S)-1a,1b-dihomo-jasmonic acid | C14H22O3 | [M+Na] |
| 0.0003283 | 261.1448 | 68.3 | Dipeptide: Glu-Leu/Ile | C11H20N2O5 | [M+H] |
| 0.0003283 | 261.1448 | 68.3 | Pantothenic acid | C9H17NO5 | [M+ACN+H] |
| 0.00010452 | 263.1398 | 69.7 | Dipeptide: Pro-Phe; Met-Leu/Ile | C14H18N2O3; C11H22N2O3S | [M+H] |
| 0.00010452 | 263.1398 | 69.7 | (3-phenylpropionyl)glycine methyl ester | C12H15NO3 | [M+ACN+H] |
| 0.00084911 | 265.1554 | 67.0 | Dipeptide: Val-Phe | C14H20N2O3 | [M+H] |
| 0.01631782 | 268.1034 | 75.3 | Adenosine; Deoxyguanosine | C10H13N5O4 | [M+H] |
| 0.00102163 | 272.9454 | 60.0 |  |  |  |
| 0.00097429 | 276.1178 | 189.4 | Dipeptide: Glu-Gln Tripeptide: Glu-Ala-Gly; Ala-Asp-Ala | C10H17N3O6 | [M+H] |
| 0.00097429 | 276.1178 | 189.4 | Dipeptide: Thr-Asp; Ser-Glu | C8H14N2O6 | [M+ACN+H] |
| 0.00595166 | 279.1709 | 66.8 | Dipeptide: Phe-Leu/Ile | C15H22N2O3 | [M+H] |
| 0.00758573 | 288.9203 | 62.8 |  |  |  |
| 0.00022987 | 294.1456 | 68.2 | Bifemelane (M6) | C17H21NO2 | [M+Na] |
| 0.00022987 | 294.1456 | 68.2 | Dipeptide: Phe-Gln Tripeptide: Phe-Ala-Gly | C14H19N3O4 | [M+H] |
| 0.00022987 | 294.1456 | 68.2 | Dipeptide: Phe-Ser; Ala-Tyr | C12H16N2O4 | [M+ACN+H] |
| 0.00069039 | 294.1827 | 57.3 | Dextromethorphan | C18H25NO | [M+Na] |
| 0.00069039 | 294.1827 | 57.3 | Dipeptide: Lys-Phe | C15H23N3O3 | [M+H] |
| 0.00040185 | 294.6906 | 61.0 |  |  |  |
| 0.01152609 | 295.1661 | 67.4 | Alpha-estradiol; beta estradiol | C18H24O2 | [M+Na] |
| 0.01152609 | 295.1661 | 67.4 | Dipeptide: Leu/Ile-Tyr | C15H22N2O4 | [M+H] |
| 0.00030829 | 299.1262 | 77.5 | 2-furanpropanoic acid; 7C-aglycone | C18H18O4 | [M+H] |
| 0.00283375 | 300.2878 | 145.8 | Amino-octadecanoic acid; palmitoylethanolamide; 3-dehydrosphinganine | C18H37NO2 | [M+H] |
| 0.00485559 | 306.1280 | 186.1 | Tripeptide: Glu-Gly-Thr; Glu-Ser-Ala; Asp-Ala-Thr | C11H19N3O7 | [M+H] |
| 4.9395E-05 | 311.1005 | 462.8 |  |  |  |
| 0.00042986 | 312.1035 | 445.8 | DIPYRONE | C13H17N3O4S | [M+H] |
| 0.00308693 | 315.1213 | 78.6 | Dihydropteroic acid; DEOXYSAPPANONE B 7,3\'-DIMETHYL ETHER | C14H14N6O3; C18H18O5 | [M+H] |
| 0.00870741 | 316.1222 | 167.4 |  |  |  |
| 3.5945E-06 | 323.1252 | 89.0 |  |  |  |
| 1.2952E-05 | 327.0528 | 63.6 |  |  |  |
| 0.00028246 | 327.1573 | 77.7 |  |  |  |
| 0.00120229 | 333.1388 | 325.5 | Tripeptide: Gln-Ala-Asp; Gly-Glu-Gln; Asn-Glu-Ala; | C12H20N4O7 | [M+H] |
| 0.00120229 | 333.1388 | 325.5 | Tripeptide: Thr-Gly-Asp; Asp-Ser-Ala; Glu-Gly-Ser | C10H17N3O7 | [M+ACN+H] |
| 0.0005433 | 340.9332 | 59.9 |  |  |  |
| 2.886E-06 | 341.1718 | 78.1 | Ubiquinone (Q2) | C19H26O4 | [M+Na] |
| 2.886E-06 | 341.1718 | 78.1 | (16b,17a)-13-ethyl-2,16,17-trihydroxy-18,19-Dinorpregna-4,9,11-trien-20-yn-3-one | C21H24O4 | [M+H] |
| 1.9076E-06 | 342.1763 | 75.4 | Tripeptide: Val-His-Ser | C14H23N5O5 | [M+H] |
| 1.9076E-06 | 342.1763 | 75.4 | Tripeptide: Pro-Gly-Gln; Ala-Pro-Asn | C12H20N4O5 | [M+ACN+H] |
| 2.5605E-06 | 356.1461 | 90.9 | Tripeptide: Val-Glu-Ser; Asp-Ser-Leu/Ile; Val-Asp-Thr | C13H23N3O7 | [M+Na] |
| 2.5605E-06 | 356.1461 | 90.9 | Tripeptide: Ser-Tyr-Ser | C15H21N3O7 | [M+H] |
| 2.5605E-06 | 356.1461 | 90.9 | Dihydropteroic acid | C14H14N6O3 | [M+ACN+H] |
| 0.00028606 | 357.1291 | 105.7 | Tripeptide: Gly-Cys-Arg; | C11H22N6O4S | [M+Na] |
| 0.00028606 | 357.1291 | 105.7 | PIOGLITAZONE | C19H20N2O3S | [M+H] |
| 0.00624222 | 357.2769 | 299.1 | Docosatrienoic acid | C22H38O2 | [M+Na] |
| 0.00624222 | 357.2769 | 299.1 | Tetracosahexaenoic acid; 5&beta;-chola-8(14),11-dien-24-oic acid | C24H36O2 | [M+H] |
| 0.00624222 | 357.2769 | 299.1 | Decanoyl-L-carnitine | C17H33NO4 | [M+ACN+H] |
| 0.00020062 | 358.1317 | 123.4 |  |  |  |
| 0.01513106 | 358.2804 | 221.1 | L-Hexanoyl carnitine n-butyl ester | C17H34NO4 | [M+ACN+H] |
| 0.01361251 | 359.1276 | 94.6 |  |  |  |
| 0.01001864 | 363.5515 | 64.6 |  |  |  |
| 0.00011419 | 366.2225 | 52.8 |  |  |  |
| 9.1066E-06 | 368.0925 | 219.1 | Tripeptide: Cys-Met-Asp | C12H21N3O6S2 | [M+H] |
| 0.00138457 | 373.0709 | 358.0 |  |  |  |
| 0.01008068 | 373.1152 | 157.7 | Tripeptide: Asn-Ser-Met; Thr-Cys-Gln | C12H22N4O6S | [M+Na] |
| 0.01008068 | 373.1152 | 157.7 | N-dealkylzuclopenthixol sulfoxide | C20H21ClN2OS | [M+H] |
| 0.00918683 | 373.1890 | 198.7 | 4-(2-morpholinoethyl)-3, 3-diphenyl-2-pyrrolidinone | C22H26N2O2 | [M+Na] |
| 0.00918683 | 373.1890 | 198.7 | Biocytin; Tripeptide: Ala-Pro-Trp | C16H28N4O4S; C19H24N4O4 | [M+H] |
| 0.00918683 | 373.1890 | 198.7 | Tripeptide: Cys-Pro-Ile/Leu | C14H25N3O4S | [M+ACN+H] |
| 0.00744303 | 373.2058 | 136.6 | Tripeptide: Lys-Glu-Pro | C16H28N4O6 | [M+H] |
| 0.00744303 | 373.2058 | 136.6 | Tripeptide: Glu-Ala-Ile/Leu; Val-Asp-Val | C14N25N3O6 | [M+ACN+H] |
| 0.01456721 | 373.6161 | 142.2 |  |  |  |
| 0.00264068 | 373.9707 | 104.3 |  |  |  |
| 0.00027006 | 374.1275 | 531.3 | Lomefloxacin | C17H19F2N3O3 | [M+Na] |
| 9.7115E-05 | 374.2169 | 53.8 | Tripeptide: Ala-Arg-Gln | C14H27N7O5 | [M+H] |
| 9.7115E-05 | 374.2169 | 53.8 | Tripeptide: Ser-Ala-Arg; Arg-Thr-Gly | C12H24N6O5 | [M+ACN+H] |
| 0.00796608 | 376.1432 | 307.5 | Tripeptide: Asn-Glu-Asn; Asn-Glu-Asp | C13H21N5O8 | [M+H] |
| 0.00796608 | 376.1432 | 307.5 | Tripeptide: Asp-Ser-Asn | C11H18N4O8 | [M+ACN+H] |
| 0.01055987 | 384.1125 | 497.7 | Tripeptide: Asn-Asp-Asn | C12H19N5O8 | [M+Na] |
| 0.01055987 | 384.1125 | 497.7 | Succinoadenosine | C14H17N5O8 | [M+H] |
| 0.00091976 | 387.2046 | 253.4 | Tripeptide: Val-Met-Pro | C15H27N3O4S | [M+ACN+H] |
| 0.00603352 | 389.2667 | 287.0 |  | C24H36O4 | [M+H] |
| 5.6669E-07 | 391.1530 | 74.7 | Testosterone sulfate; PA (6:0/6:0) | C19H28O5S; C15H29O8P | [M+Na] |
| 0.00878819 | 394.1248 | 417.7 | Tripeptide: Met-Glu-Asp | C14H23N3O8S | [M+H] |
| 0.00878819 | 394.1248 | 417.7 | Tripeptide: Glu-Cys-Cys | C11H20N4O5S2 | [M+ACN+H] |
| 0.00025623 | 395.0394 | 93.5 |  |  |  |
| 0.00171646 | 398.0666 | 553.5 |  |  |  |
| 3.2925E-05 | 407.2289 | 68.6 | Tripeptide: Pro-Tyr-Lys; Gln-Phe-Leu/Ile | C20H30N4O5 | [M+H] |
| 3.2925E-05 | 407.2289 | 68.6 | Tripeptide: Tyr-Ala-Ile/Leu; Ile/Leu-Ser-Phe | C18H27N3O5 | [M+ACN+H] |
| 0.00056533 | 408.9206 | 60.5 |  |  |  |
| 0.00077706 | 415.1181 | 220.7 | Dihydrorotenone | C23H20O6 | [M+Na] |
| 0.00283645 | 416.1371 | 157.6 | N-desmethyltrifluoperazine | C20H22F3N3S | [M+Na] |
| 0.00945991 | 419.0387 | 545.3 |  |  |  |
| 0.00492455 | 419.0387 | 178.2 |  |  |  |
| 0.00079019 | 419.2348 | 58.0 | Tripeptide: Ile/Leu-Lys-His | C18H23N6O4 | [M+Na] |
| 6.2036E-05 | 419.4353 | 57.1 |  |  |  |
| 8.6927E-06 | 419.6362 | 56.0 |  |  |  |
| 0.00703314 | 420.2562 | 172.1 |  |  |  |
| 0.00036925 | 426.1661 | 457.7 | Tripeptide: Glu-Met-Phe | C19H27N3O6S | [M+H] |
| 0.00029885 | 427.0915 | 167.4 | Cysteine-Glutathione disulfide | C13H22N4O8S2 | [M+H] |
| 0.00591962 | 427.1706 | 443.7 |  |  |  |
| 0.01060251 | 429.2153 | 369.0 |  |  |  |
| 0.01652339 | 431.4779 | 54.9 |  |  |  |
| 0.01372402 | 432.1685 | 104.7 | Tripeptide: Glu-Glu-Asn; Asp-Gln-Glu | C14H22N4O9 | [M+ACN+H] |
| 0.0002753 | 433.1747 | 83.1 | Quinaprilat | C23H26N2O5 | [M+Na] |
| 9.5792E-11 | 437.5655 | 56.0 |  |  |  |
| 1.0649E-07 | 437.8997 | 56.6 |  |  |  |
| 7.6476E-05 | 438.2282 | 73.0 |  |  |  |
| 0.00143328 | 441.4847 | 54.3 |  |  |  |
| 0.00221002 | 441.7358 | 54.4 |  |  |  |
| 3.2667E-06 | 442.7580 | 59.7 |  |  |  |
| 1.7087E-07 | 447.2471 | 116.5 | Tripeptide: His-Arg-Ile/Leu | C18H32N8O4 | [M+Na] |
| 1.7087E-07 | 447.2471 | 116.5 | Tripeptide: Lys-Cys-Arg; Ile/Leu-His-His | C15H31N7O4S; C18H27N7O4 | [M+ACN+H] |
| 3.3759E-06 | 447.4980 | 55.8 |  |  |  |
| 0.01307328 | 448.1482 | 453.5 | Tripeptide: Met-Glu-Phe | C19H27N3O6S | [M+Na] |
| 0.01222109 | 448.1695 |  | desmethylnortriptyline glucuronide | C24H27NO6 | [M+Na] |
| 2.1446E-10 | 448.7591 |  |  |  |  |
| 5.8009E-11 | 449.0099 |  |  |  |  |
| 0.00793343 | 449.1812 |  | Tripeptide: Phe-Phe-Asn | C22H26N4O5 | [M+Na] |
| 1.0492E-05 | 456.1686 |  | Tripeptide: Phe-Met-His | C20H27N5O4S | [M+Na] |
| 0.00296679 | 458.0139 |  |  |  |  |
| 9.9907E-05 | 458.2662 |  |  |  |  |
| 8.8199E-05 | 460.2009 |  | Monodeallyalmitrine | C23H25F2N7 | [M+Na] |
| 0.00107529 | 460.2659 | 59.1 | Tripeptide: Arg-Arg-Glu; Trp-Val-Arg | C17H33N9O6; C22H33N7O4 | [M+H] |
| 0.00107529 | 460.2659 | 59.1 | Tripeptide: Phe-Arg-Pro; Met-Arg-Ile/Leu | C20H30N6O4; C17H34N6O4S | [M+ACN+H] |
| 1.4564E-05 | 461.2053 |  |  |  |  |
| 4.6711E-06 | 462.1796 |  |  |  |  |
| 4.7624E-05 | 463.0274 |  |  |  |  |
| 0.00051465 | 465.7436 |  |  |  |  |
| 0.00046969 | 465.9946 |  |  |  |  |
| 0.00158165 | 466.2445 | 56.2 | Tripeptide: Arg-Gln-Tyr | C20H31N7O6 | [M+H] |
| 0.00158165 | 466.2445 | 56.2 | Tripeptide: Ser-Arg-Tyr; Met-Phe-Lys | C18H28N6O6; C20H32N4O4S | [M+ACN+H] |
| 0.00050946 | 467.1803 | 81.2 | Tripeptide: Met-Trp-Met | C21H30N4O4S2 | [M+H] |
| 0.00050946 | 467.1803 | 81.2 | Tripeptide: Asp-Tyr-Glu | C18H23N3O9 | [M+ACN+H] |
| 3.9803E-05 | 470.1066 |  |  |  |  |
| 1.3059E-07 | 473.3236 |  |  |  |  |
| 0.002501 | 474.3269 |  |  |  |  |
| 4.1777E-07 | 475.2196 |  |  |  |  |
| 0.00109518 | 476.9085 |  |  |  |  |
| 0.00414098 | 477.1757 |  | Tripeptide: Ser-Trp-Tyr | C23H26N4O6 | [M+Na] |
| 0.00017733 | 477.9098 |  |  |  |  |
| 1.2873E-06 | 478.9379 |  |  |  |  |
| 1.7679E-06 | 479.0194 |  |  |  |  |
| 3.8289E-08 | 479.2705 |  |  |  |  |
| 1.0056E-05 | 479.5208 |  |  |  |  |
| 5.8281E-05 | 483.5037 |  |  |  |  |
| 4.4791E-05 | 483.7533 |  |  |  |  |
| 6.4549E-06 | 485.2922 |  | PG (16:0/0:0) [U] | C22H45O9P | [M+H] |
| 0.0001213 | 485.3238 |  |  |  |  |
| 2.4567E-05 | 485.7930 |  |  |  |  |
| 5.6008E-05 | 487.9060 |  |  |  |  |
| 2.2898E-06 | 489.1921 |  |  |  |  |
| 0.00027232 | 489.2275 |  | Tripeptide: Trp-Trp-Gly | C24H25N5O4 | [M+ACN+H] |
| 0.00662046 | 489.3126 |  |  |  |  |
| 8.4025E-05 | 490.3494 |  | SM (d18:0/0:0) | C23H52N2O5P | [M+Na] |
| 8.4025E-05 | 490.3494 |  | 1&alpha; 23R, 25S, 26-tetrahydroxy vitamin D3 | C27H44O5 | [M+ACN+H] |
| 1.2666E-06 | 491.5170 |  |  |  |  |
| 6.0779E-07 | 491.7673 |  |  |  |  |
| 1.9068E-06 | 492.0180 |  |  |  |  |
| 7.8011E-07 | 492.1735 |  |  |  |  |
| 1.4836E-06 | 492.2696 |  |  |  |  |
| 0.00796438 | 497.1299 |  |  |  |  |
| 0.00916129 | 498.6204 |  |  |  |  |
| 0.00031057 | 501.3181 |  | PC (O-16:2(9E,10E)/0:0) [U] | C24H49NO6P | [M+Na] |
| 2.3369E-07 | 502.2865 |  | PE (18:1(9Z)/0:0) | C23H46NO7P | [M+Na] |
| 0.00125909 | 502.3491 |  | (24R)-1-Alpha; 24,25,26-tetrahydroxy vitamin D3 | C28H44O5 | [M+ACN+H] |
| 0.00012512 | 503.3331 |  | PC (O-16:1/0:0) | C24H51NO6P | [M+Na] |
| 0.0009304 | 504.3369 |  |  |  |  |
| 0.00047234 | 506.3436 |  | 11&alpha; -(chloromethyl)-1-alpha; 25-dihydroxy vitamin D3 |  |  |
| 3.5832E-07 | 508.2013 | 82.6 | b-D-Glucopyranosiduronic acid | C25H30FNO9 | [M+H] |
| 3.5832E-07 | 508.2013 | 82.6 | Tripeptide: Trp-Met-Met | C21H30N4O4S2 | [M+ACN+H] |
| 0.00044645 | 509.2085 |  |  |  |  |
| 0.01288731 | 509.2686 |  |  |  |  |
| 0.00087641 | 509.5173 |  |  |  |  |
| 0.00203008 | 512.2923 |  |  |  |  |
| 1.5885E-07 | 516.6907 |  |  |  |  |
| 0.00030072 | 516.9467 |  |  |  |  |
| 0.00505163 | 518.3214 | 308.2 | PC (16:0/0:0) | C24H50NO7P | [M+Na] |
| 0.00505163 | 518.3214 | 308.2 | Linolenoyl lysolecithin | C26H48NO7P | [M+H] |
| 0.01174926 | 518.3442 |  |  |  |  |
| 0.01018815 | 519.3255 |  | PC (16:0/0:0) | C24H51NO7P | [M+Na] |
| 5.1667E-06 | 519.9369 |  |  |  |  |
| 6.3856E-06 | 520.2720 |  |  |  |  |
| 0.00083226 | 520.6063 |  |  |  |  |
| 0.00038784 | 521.8992 |  |  |  |  |
| 2.6285E-05 | 523.5406 | 56.6 | Ceroplastic acid | C35H70O2 | [M+H] |
| 4.6891E-05 | 523.7910 |  |  |  |  |
| 5.7268E-05 | 524.0427 |  |  |  |  |
| 8.6907E-05 | 524.2935 |  | PS (18:1 (9Z)/0:0) | C24H46NO9P | [M+H] |
| 0.00031615 | 524.5448 |  |  |  |  |
| 0.0084301 | 525.3145 |  |  |  |  |
| 0.00064844 | 526.9605 |  |  |  |  |
| 0.00018784 | 527.2949 |  |  |  |  |
| 0.0067096 | 527.6370 |  |  |  |  |
| 4.8996E-05 | 528.2384 | 56.3 | Tripeptide: Trp-His-Trp | C28H29N7O4 | [M+H] |
| 0.00159628 | 528.5720 |  |  |  |  |
| 0.0002893 | 529.8870 |  |  |  |  |
| 5.6429E-05 | 531.0148 |  |  |  |  |
| 0.00238715 | 534.6383 |  |  |  |  |
| 0.00023452 | 534.8955 |  |  |  |  |
| 2.6055E-05 | 537.5375 |  |  |  |  |
| 2.2352E-07 | 537.7892 |  |  |  |  |
| 0.0033242 | 537.8736 |  |  |  |  |
| 1.1875E-05 | 542.3008 |  |  |  |  |
| 0.00095016 | 542.5019 |  |  |  |  |
| 1.6076E-05 | 542.7025 |  |  |  |  |
| 0.00024884 | 544.8968 |  |  |  |  |
| 0.01133224 | 552.1570 |  |  |  |  |
| 0.00569428 | 555.8931 |  |  |  |  |
| 0.00583878 | 557.3081 |  |  |  |  |
| 9.2922E-06 | 557.7991 |  |  |  |  |
| 9.8196E-05 | 563.8799 |  |  |  |  |
| 0.00015866 | 564.9754 |  |  |  |  |
| 1.7898E-05 | 565.3083 |  | PC (20:5 (5Z,8Z,11Z,14Z,17Z)/0:0) | C28H49NO7P | [M+Na] |
| 0.00019519 | 565.6456 |  |  |  |  |
| 0.00136169 | 574.9680 |  |  |  |  |
| 0.00358003 | 575.3022 |  | Triterpenoid | C30H48O7S | [M+Na] |
| 0.01234725 | 581.2211 |  |  |  |  |
| 0.00040859 | 589.8876 |  |  |  |  |
| 2.5688E-06 | 595.6590 |  |  |  |  |
| 1.0132E-06 | 595.9926 |  |  |  |  |
| 7.1483E-05 | 597.8738 |  |  |  |  |
| 0.00262582 | 599.0024 |  |  |  |  |
| 0.00049173 | 605.8614 |  |  |  |  |
| 6.6667E-07 | 612.3238 | 54.8 | Dihydroergocristine | C35H41N5O5 | [M+H] |
| 2.0662E-06 | 612.6566 |  |  |  |  |
| 0.00119259 | 612.8828 |  |  |  |  |
| 5.7451E-06 | 612.9910 |  |  |  |  |
| 0.00183288 | 617.3171 |  |  |  |  |
| 0.00030202 | 621.2496 |  |  |  |  |
| 0.00289496 | 624.2826 |  | Biliverdin IX | C33H37N5O5 | [M+ACN+H] |
| 9.2901E-08 | 628.3789 |  |  |  |  |
| 0.00700768 | 628.8577 |  |  |  |  |
| 0.00277446 | 631.8669 |  |  |  |  |
| 0.00271078 | 639.8529 |  |  |  |  |
| 0.0002906 | 645.3610 |  |  |  |  |
| 0.00041795 | 645.6124 |  |  |  |  |
| 1.0889E-06 | 655.0212 |  |  |  |  |
| 8.9168E-07 | 655.3547 |  |  |  |  |
| 3.213E-08 | 655.6899 |  |  |  |  |
| 1.6506E-10 | 656.0238 |  |  |  |  |
| 0.001406 | 665.2837 |  |  |  |  |
| 0.00233103 | 666.3717 |  |  |  |  |
| 0.01140626 | 673.8464 |  |  |  |  |
| 0.00097069 | 680.8716 |  |  |  |  |
| 0.00643226 | 681.4923 |  |  |  |  |
| 3.199E-05 | 697.7184 |  |  |  |  |
| 0.00053743 | 698.0539 |  |  |  |  |
| 0.0119141 | 698.3850 |  |  |  |  |
| 0.0013357 | 699.8548 |  |  |  |  |
| 0.00118225 | 748.8601 |  |  |  |  |
| 0.01156314 | 778.2895 |  |  |  |  |
| 0.00034615 | 779.3993 |  |  |  |  |

B.

| **p-value** | **m/z** | **RT** | **Identity** | **Formula** | **Adduct** |
| --- | --- | --- | --- | --- | --- |
| 1.07E-07 | 132.1013 | **103.1** | Leucine/Isoleucine | C6H13NO2 | [M+H] |
| 1.52E-02 | 148.0598 | **104.4** | Glutamate | C5H9NO4 | [M+H] |
| 7.03E-06 | 150.0577 | **106.7** | Methionine | C5H11NO2S | [M+H] |
| 9.68E-03 | 157.1218 | **407.6** | 4-hydroxy Nonenal; 2E-nonenoic acid | C9H16O2 | [M+H] |
| 9.27E-07 | 166.0854 | **104.6** | Phenylalanine | C9H11NO2 | [M+H] |
| 1.92E-03 | 168.0683 |  |  |  |  |
| 3.03E-06 | 182.0803 | **105.7** | Tyrosine | C9H11NO3 | [M+H] |
| 3.03E-06 | 182.0803 | **105.7** | 3-Dehydrocarnitine | C7H13NO3 | [M+Na] |
| 1.37E-04 | 189.1230 | **104.6** | Dipeptide: Gly-Ile/Leu; Val-Ala; Acetyl Lysine | C8H16N2O3 | [M+H] |
| 3.47E-03 | 196.0995 |  |  |  |  |
| 2.18E-03 | 203.1384 | **105.8** | Dipeptide: Ala-Ile/Leu | C9H18N2O3 | [M+H] |
| 2.18E-03 | 203.1384 | **105.8** | Carnitine | C7H15NO3 | [M+ACN+H] |
| 2.08E-05 | 205.0961 | **101.8** | Tryptophan | C11H12N2O2 | [M+H] |
| 2.00E-04 | 210.1150 |  |  |  |  |
| 1.19E-05 | 211.1185 | **107.0** | N,N-Dimethyltryptamine (DMT) | C12H16N2 | [M+Na] |
| 1.19E-05 | 211.1185 | **107.0** | 1-methylhistidine | C7H11N3O2 | [M+ACN+H] |
| 1.84E-04 | 212.1111 |  |  |  |  |
| 2.58E-03 | 219.1333 | **103.9** | Dipeptide: Ser-Ile/Leu; Val-Thr | C9H18N2O4 | [M+H] |
| 5.60E-03 | 221.0424 | **98.9** | Vanillylmandelic acid | C9H10O5 | [M+Na] |
| 2.39E-03 | 228.0716 |  |  |  |  |
| 1.25E-05 | 229.1540 | **103.7** | Dipeptide: Pro-Ile/Leu | C11H20N2O3 | [M+H] |
| 1.25E-05 | 229.1540 | **103.7** | 8-Amino-7-oxononanoate | C9H17NO3 | [M+ACN+H] |
| 2.68E-04 | 231.1701 | **103.8** | Dipeptide: Val-Ile/Leu | C11H22N2O3 | [M+H] |
| 2.68E-04 | 231.1701 | **103.8** | 5,8-tetradecadienal | C14H24O | [M+Na] |
| 1.98E-03 | 233.1488 | **105.1** | Dipeptide: Thr-Ile/Leu | C10H20N2O4 | [M+H] |
| 1.17E-05 | 244.0993 |  |  |  |  |
| 8.70E-05 | 245.1851 | **106.1** | Dipeptide:Ile/Leu-Ile/Leu | C12H24N2O3 | [M+H] |
| 9.41E-05 | 258.1082 | **104.9** | 5-Methylcytidine; Glycerophosphocholine | C10H15N3O5; C8H20NO6P | [M+H] |
| 5.70E-05 | 260.0942 |  |  |  |  |
| 1.65E-03 | 260.1601 | **103.2** | Dipeptide: Gln-Ile/Leu Tripeptide: Val-Ala-Ala; Ala-Gly-Ile/Leu | C11H21N3O4 | [M+H] |
| 1.65E-03 | 260.1601 | **103.2** | Dipeptide: Ile/Leu-Ser; Val-Thr | C9H18N2O4 | [M+ACN+H] |
| 1.27E-03 | 261.1442 | **105.5** | Dipeptide: Glu-Ile/Leu | C11H20N2O5 | [M+H] |
| 1.27E-03 | 261.1442 | **105.5** | (9R,13R)-1a,1b-dihomo-jasmonic acid | C14H22O3 | [M+Na] |
| 1.27E-03 | 261.1442 | **105.5** | Pantothenic Acid | C9H17NO5 | [M+ACN+H] |
| 9.75E-03 | 263.1405 | **106.5** | Dipeptide: Pro-Phe; Met-Ile/Leu | C14H18N2O3; C11H22N2O3S1 | [M+H] |
| 4.56E-04 | 265.1539 | **105.0** | Dipeptide: Val-Phe | C14H20N2O3 | [M+H] |
| 1.49E-02 | 268.1024 | **103.6** | Neuraminic acid; Adenosine; Deoxyguanosine | C9H17NO8; C10H13N5O4; | [M+H] |
| 1.35E-03 | 269.0864 | **110.5** | 3-Deoxy-D-glycero-D-galacto-2-nonulosonic acid; Inosine | C9H16O9; C10H12N4O5 | [M+H] |
| 1.35E-03 | 269.0864 | **110.5** | Tripeptide: Gly-Asn-Gly Dipeptide: Asn-Asn | C8H14N4O5 | [M+Na] |
| 1.52E-02 | 269.2827 | **317.5** | Stearaldehyde | C18H36O | [M+H] |
| 7.30E-03 | 277.0638 |  |  |  |  |
| 3.16E-03 | 279.1692 | **106.8** | Dipeptide: Ile/Leu-Phe | C15H22N2O3 | [M+H] |
| 1.47E-05 | 280.0944 | **98.7** | Tripeptide: Thr-Cys-Gly; Ala-Cys-Ser | C9H17N3O5S1 | [M+H] |
| 1.47E-05 | 280.0944 | **98.7** | Glycerophosphocholine | C8H20NO6P | [M+Na] |
| 1.47E-05 | 280.0944 | **98.7** | Cystathionine sulfoxide | C7H14N2O5S | [M+ACN+H] |
| 6.96E-04 | 283.1104 |  |  |  |  |
| 1.18E-02 | 295.1648 | **104.4** | Dipeptide: Tyr-Ile/Leu | C15H22N2O4 | [M+H] |
| 1.18E-02 | 295.1648 | **104.4** | B-estradiol | C18H24O2 | [M+Na] |
| 1.29E-02 | 298.2730 | **474.8** | 3-ketosphingosine | C18H35NO2 | [M+H] |
| 1.29E-02 | 298.2730 | **474.8** | Palmitic acid | C16H32O2 | [M+ACN+H] |
| 6.75E-06 | 299.1252 | **103.9** | 7-Methylguanosine | C11H16N5O5 | [M+H] |
| 6.43E-03 | 300.2887 | **491.3** | Palmitoylethanolamide; Sphingosine; 3-ketosphinganine | C18H37NO2 | [M+H] |
| 1.91E-04 | 301.2921 |  |  |  |  |
| 1.68E-06 | 302.3043 | **452.1** | Sphinganine | C18H39NO2 | [M+H] |
| 7.75E-07 | 315.1204 | **104.1** | Dihydropteroic acid | C14H14N6O3 | [M+H] |
| 7.75E-07 | 315.1204 | **104.1** | Dipeptide: His-His | C12H16N6O3 | [M+Na] |
| 3.19E-03 | 315.1399 |  |  |  |  |
| 1.07E-04 | 323.1248 |  |  |  |  |
| 6.05E-06 | 327.1561 |  |  |  |  |
| 1.19E-04 | 332.1244 | **105.8** | Dipeptide: Gln-Tyr Tripeptide: Tyr-Gly-Ala; Ser-Gly-Phe | C14H19N3O5 | [M+Na] |
| 1.03E-04 | 333.1277 |  |  |  |  |
| 3.56E-07 | 341.1712 | **102.4** | Ubiquinone (Q2) | C19H26O4 | [M+Na] |
| 1.11E-07 | 342.1752 | **103.5** | Tripeptide: His-Ser-Val | C14H23N5O5 | [M+H] |
| 1.11E-07 | 342.1752 | **103.5** | Tripeptide: Pro-Gly-Gln; Ala-Pro-Asn | C12H20N4O5 | [M+ACN+H] |
| 9.45E-05 | 346.1155 | **105.0** | Tripeptide: His-Cys-Ser | C12H19N5O5S1 | [M+H] |
| 4.00E-12 | 356.1458 | **103.4** | Tripeptide: Ser-Tyr-Ser | C15H21N3O7 | [M+H] |
| 4.00E-12 | 356.1458 | **103.4** | Tripeptide: Asp-Val-Thr; Ile/Leu-Ser-Asp; Glu-Ser-Val | C13H23N3O7 | [M+Na] |
| 4.00E-12 | 356.1458 | **103.4** | Dihydropteroic acid | C14H14N6O3 | [M+ACN+H] |
| 5.86E-04 | 356.1858 |  |  |  |  |
| 5.01E-05 | 357.1299 | **103.7** | Tripeptide: Arg-Gly-Cys | C11H22N6O4S1 | [M+Na] |
| 8.08E-03 | 357.2769 | **470.3** | tetracosahexaenoic acid | C24H36O2 | [M+H] |
| 8.08E-03 | 357.2769 | **470.3** | Docosatrienoic acid | C22H38O2 | [M+Na] |
| 8.08E-03 | 357.2769 | **470.3** | Decanoyl-L-carnitine | C17H33NO4 | [M+ACN+H] |
| 9.12E-07 | 359.1279 |  |  |  |  |
| 1.23E-02 | 359.3674 |  |  |  |  |
| 6.54E-03 | 373.1153 | **127.4** | Tripeptide: Ser-Met-Asn; Gln-Thr-Cys | C12H22N4O6S1 | [M+Na] |
| 1.12E-04 | 373.9658 |  |  |  |  |
| 2.55E-04 | 374.2167 | **90.3** | Tripeptide: Ala-Gln-Arg | C14H27N7O5 | [M+H] |
| 2.55E-04 | 374.2167 | **90.3** | Tripeptide: Arg-Ser-Ala; Thr-Gly-Arg Dipeptide: Lys-Thr | C12H24N6O5 | [M+ACN+H] |
| 9.38E-03 | 374.4676 |  |  |  |  |
| 6.46E-07 | 375.1554 |  |  |  |  |
| 7.84E-05 | 376.1589 | **104.6** | Tripeptide: Asn-Trp-Gly; Gly-Tyr-His | C17H21N5O5 | [M+H] |
| 7.84E-05 | 376.1589 | **104.6** | Tripeptide: Thr-Pro-His | C15H23N5O5 | [M+Na] |
| 1.32E-02 | 387.1460 | **107.9** | Tripeptide: His-Cys-Gln | C14H22N6O5S1 | [M+H] |
| 1.32E-02 | 387.1460 | **107.9** | Tripeptide: Cys-His-Ser | C12H19N5O5S1 | [M+ACN+H] |
| 6.35E-03 | 388.1557 |  |  |  |  |
| 6.81E-03 | 389.2671 | **481.6** | 7a-Hydroxy-3-oxo-4-cholenoic acid | C24H36O4 | [M+H] |
| 9.16E-04 | 391.1530 | **98.6** | PA(6:0/6:0) | C15H29O8P | [M+Na] |
| 9.16E-04 | 391.1530 | **98.6** | Testosterone sulfate | C19H28O5S | [M+Na] |
| 4.59E-07 | 396.1778 | **105.0** | Tripeptide: Thr-Glu-Phe; Asp-Tyr-Val | C18H25N3O7 | [M+H] |
| 3.25E-06 | 398.1935 |  |  |  |  |
| 3.11E-06 | 407.2273 | **106.2** | Tripeptide: Pro-Tyr-Lys; Phe-Gln-Ile/Leu | C20H30N4O5 | [M+H] |
|  |  |  | Tripeptide: Tyr |  |  |
| 6.42E-03 | 412.1740 | **104.8** | Tripeptide: Thr-Tyr-Glu | C18H25N3O8 | [M+H] |
| 6.42E-03 | 412.1740 | **104.8** | Tripeptide: His-His-Pro | C17H23N7O4 | [M+Na] |
| 1.12E-04 | 412.2095 | **102.4** | PE(6:0/6:0) | C17H34NO8P | [M+H] |
| 1.74E-02 | 415.1412 | **100.3** | Tripeptide: Gly-Met-Trp | C18H24N4O4S1 | [M+Na] |
| 9.13E-03 | 416.1361 |  |  |  |  |
| 1.94E-05 | 419.0355 |  |  |  |  |
| 3.81E-05 | 419.2348 | **105.5** | Tripeptide: His-Lys-Ile/Leu | C18H32N6O4 | [M+Na] |
| 2.12E-03 | 419.4356 |  |  |  |  |
| 4.38E-04 | 419.6366 |  |  |  |  |
| 1.33E-03 | 424.2097 | **105.0** | Tripeptide: Ile/Leu-Tyr-Glu | C20H29N3O7 | [M+H] |
| 1.53E-02 | 428.0340 | **246.3** | ADP; Deoxyguanosine diphosphate (dGDP); Adenosine 3\',5\'-bisphosphate (PAP) | C10H15N5O10P2 | [M+H] |
| 7.99E-05 | 428.2040 | **102.5** | Tripeptide: His-His-Ile/Leu; Arg-Lys-Cys | C18H27N7O4; C15H31N7O4S1 | [M+Na] |
| 6.53E-03 | 430.1193 |  |  |  |  |
| 1.41E-05 | 432.1690 |  |  |  |  |
| 4.13E-07 | 437.5667 |  |  |  |  |
| 7.44E-10 | 437.9017 |  |  |  |  |
| 1.13E-05 | 438.2250 |  |  |  |  |
| 2.61E-04 | 439.2310 | **103.9** | Tripeptide: Arg-Thr-Tyr | C19H30N6O6 | [M+H] |
| 2.61E-04 | 439.2310 | **103.9** | Tripeptide: Glu-Arg-Ile/Leu; Trp-Val-Ile/Leu | C22H32N4O4 | [M+Na] |
| 6.05E-04 | 440.2416 |  |  |  |  |
| 2.86E-04 | 441.4869 |  |  |  |  |
| 1.58E-04 | 441.7369 |  |  |  |  |
| 5.63E-06 | 442.7586 |  |  |  |  |
| 9.64E-03 | 443.5595 |  |  |  |  |
| 1.34E-02 | 443.8946 |  |  |  |  |
| 1.48E-02 | 446.1943 | **104.6** | Tripeptide: Tyr-Thr-Tyr | C22H27N3O7 | [M+H] |
| 2.66E-03 | 446.9974 |  |  |  |  |
| 5.02E-10 | 447.2473 | **93.1** | Tripeptide: His-Arg-Ile/Leu; | C18H32N8O4 | [M+Na] |
| 1.72E-07 | 447.4995 |  |  |  |  |
| 8.25E-09 | 448.7598 |  |  |  |  |
| 9.39E-10 | 449.0112 |  |  |  |  |
| 1.89E-02 | 449.1825 | **103.4** | Tripeptide: Phe-Asn-Phe; His-Asp-Arg | C22H26N4O5; C16H26N8O6 | [M+Na] |
| 5.47E-11 | 449.2612 |  |  |  |  |
| 6.42E-03 | 454.2574 |  |  |  |  |
| 1.79E-02 | 457.3278 | **449.7** | (22S)-1&alpha;,22,25-trihydroxy-26,27-dimethyl-23,23,24,24-tetradehydrovitamin D3 / (22S)-1&alpha;,22,25-trihydroxy-26,27-dimethyl-23,23,24,24-tetradehydrocholecalciferol | C29H44O4 | [M+H] |
| 1.79E-02 | 457.3278 | **449.7** | 3a,7a-Dihydroxycoprostanic acid | C27H46O4 | [M+Na] |
| 9.99E-06 | 457.3354 |  |  |  |  |
| 1.85E-04 | 458.0164 |  |  |  |  |
| 7.36E-05 | 458.2680 |  |  |  |  |
| 2.35E-05 | 460.2001 |  |  |  |  |
| 3.52E-06 | 460.2653 | **89.3** | Tripeptide: Arg-Arg-Glu; Trp-Val-Arg | C17H33N9O6; C22H33N7O4 | [M+H] |
| 2.67E-06 | 462.1794 |  |  |  |  |
| 2.92E-03 | 465.7445 |  |  |  |  |
| 1.73E-04 | 465.9958 | **90.2** | Adenosine 5-O-(3-thiodiphophate) | C10H15N5O9P2S | [M+Na] |
| 4.97E-04 | 466.2451 | **90.1** | Tripeptide: Tyr-Arg-Gln | C20H31N7O6 | [M+H] |
| 4.73E-03 | 467.1783 | **108.2** | Tripeptide: Met-Trp-Met | C21H30N4O4S2 | [M+H] |
| 4.25E-03 | 469.2303 |  |  |  |  |
| 1.81E-02 | 469.3282 |  |  |  |  |
| 8.33E-03 | 470.1600 | **102.3** | Tripeptide: Trp-Asp-Gln; His-Glu-Tyr; Glu-Trp-Asn | C20H25N5O7 | [M+Na] |
| 6.34E-03 | 470.2149 | **106.0** | Tripeptide: Gln-Trp-His | C22H27N7O5 | [M+H] |
| 6.34E-03 | 470.2149 | **106.0** | Tripeptide: Trp-Arg-Ser | C20H29N7O5 | [M+Na] |
| 1.52E-02 | 471.5225 |  |  |  |  |
| 4.21E-10 | 473.3241 | **485.8** | Coprocholic acid | C27H46O5 | [M+Na] |
| 8.64E-08 | 474.2157 |  |  |  |  |
| 1.54E-08 | 474.3285 |  |  |  |  |
| 4.84E-07 | 475.2189 |  |  |  |  |
| 4.85E-03 | 477.1735 | **103.2** | Tripeptide: Tyr-Ser-Trp | C23H26N4O6 | [M+Na] |
| 3.57E-04 | 478.1769 | **106.6** | Tripeptide: Trp-His-Asn; His-His-Tyr | C21H25N7O5 | [M+Na] |
| 3.57E-04 | 478.1769 | **106.6** | PS(6:0/6:0) | C18H34NO10P | [M+Na] |
| 4.80E-09 | 478.9394 |  |  |  |  |
| 2.29E-05 | 479.0206 |  |  |  |  |
| 7.87E-07 | 479.2717 |  |  |  |  |
| 1.38E-04 | 479.5223 |  |  |  |  |
| 5.57E-06 | 483.5036 |  |  |  |  |
| 1.08E-06 | 483.7542 |  |  |  |  |
| 2.41E-06 | 485.2916 | **90.1** | PG(16:0/0:0)[U] | C22H45O9P | [M+H] |
| 2.77E-05 | 485.3235 |  |  |  |  |
| 2.22E-04 | 486.3269 |  |  |  |  |
| 5.91E-03 | 487.3389 | **397.2** | Trihydroxycoprostanoic acid | C28H48O5 | [M+Na] |
| 1.23E-02 | 488.2405 |  |  |  |  |
| 1.59E-03 | 489.1686 |  |  |  |  |
| 1.13E-05 | 489.1895 |  |  |  |  |
| 3.07E-03 | 489.2286 | **105.9** | Tripeptide: His-Trp-Phe | C26H28N6O4 | [M+H] |
| 6.61E-08 | 489.3180 | **386.6** | Varanic acid | C27H46O6 | [M+Na] |
| 6.61E-08 | 489.3180 | **386.6** | PC(P-15:0/0:0) | C23H49NO6P | [M+Na] |
| 1.31E-08 | 490.3497 | **471.6** | SM(d18:0/0:0) | C23H52N2O5P | [M+Na] |
| 5.35E-08 | 491.3531 |  |  |  |  |
| 1.51E-07 | 491.5173 |  |  |  |  |
| 1.10E-07 | 491.7666 |  |  |  |  |
| 1.17E-06 | 492.0187 |  |  |  |  |
| 4.09E-06 | 492.1723 |  |  |  |  |
| 7.83E-06 | 492.2699 |  |  |  |  |
| 2.07E-03 | 498.6213 |  |  |  |  |
| 8.38E-03 | 498.9555 |  |  |  |  |
| 1.12E-05 | 501.3185 | **484.9** | PC(O-16:2(9E,10E)/0:0)[U] | C24H49NO6P | [M+Na] |
| 3.05E-06 | 502.3216 |  |  |  |  |
| 1.07E-04 | 502.3489 |  |  |  |  |
| 9.60E-06 | 503.3339 | **434.8** | PC(O-16:1(11Z)/0:0) | C24H51NO6P | [M+Na] |
| 3.18E-05 | 504.3370 |  |  |  |  |
| 4.04E-07 | 506.3438 |  |  |  |  |
| 1.62E-02 | 507.3483 |  |  |  |  |
| 6.77E-07 | 508.1997 |  |  |  |  |
| 9.43E-03 | 509.2701 |  |  |  |  |
| 3.70E-03 | 509.5193 |  |  |  |  |
| 7.87E-03 | 512.2928 |  |  |  |  |
| 2.23E-03 | 518.3220 | **496.7** | PC(18:3(9Z,12Z,15Z)/0:0)[U] | C26H48NO7P | [M+H] |
| 2.23E-03 | 518.3220 | **496.7** | PC(16:0/0:0)[U] / PC(16:0/0:0)[rac] | C24H50NO7P | [M+Na] |
| 2.20E-05 | 518.3444 |  |  |  |  |
| 1.43E-02 | 519.3283 | **226.6** | PC(18:3(6Z,9Z,12Z)/0:0)[U] | C26H49NO7P | [M+H] |
| 1.43E-02 | 519.3283 | **226.6** | PC(O-14:0/2:0) | C24H51NO7P | [M+Na] |
| 1.10E-05 | 519.3482 |  |  |  |  |
| 3.19E-04 | 519.9374 |  |  |  |  |
| 2.68E-04 | 520.2724 |  |  |  |  |
| 1.73E-04 | 520.3595 |  |  |  |  |
| 1.07E-05 | 520.6072 |  |  |  |  |
| 3.28E-03 | 521.3638 |  |  |  |  |
| 1.35E-02 | 522.2962 |  |  |  |  |
| 9.67E-03 | 523.2024 |  |  |  |  |
| 2.43E-03 | 523.3004 |  |  |  |  |
| 1.74E-05 | 523.5405 |  |  |  |  |
| 6.19E-05 | 523.7909 |  |  |  |  |
| 1.45E-03 | 524.0441 |  |  |  |  |
| 1.72E-02 | 524.2954 | **90.7** | PS(18:1(9Z)/0:0) | C24H46NO9P | [M+H] |
| 4.04E-03 | 524.5467 |  |  |  |  |
| 3.63E-04 | 525.3148 |  |  |  |  |
| 7.92E-03 | 526.3192 |  |  |  |  |
| 6.54E-05 | 526.9615 |  |  |  |  |
| 4.32E-06 | 527.2986 |  |  |  |  |
| 4.16E-05 | 528.2381 | **89.5** | Tripeptide: Trp-His-Trp | C28H29N7O4 | [M+H] |
| 2.61E-05 | 537.5393 |  |  |  |  |
| 3.98E-05 | 537.7896 |  |  |  |  |
| 7.47E-05 | 542.3016 |  |  |  |  |
| 1.13E-04 | 542.5032 |  |  |  |  |
| 1.76E-05 | 542.7039 |  |  |  |  |
| 5.77E-04 | 542.9044 |  |  |  |  |
| 5.48E-04 | 548.8307 |  |  |  |  |
| 1.84E-02 | 549.1968 |  |  |  |  |
| 1.59E-05 | 564.9763 |  |  |  |  |
| 1.51E-04 | 565.3105 | **90.5** | PC(20:5(5Z,8Z,11Z,14Z,17Z)/0:0) | C28H49NO7P | [M+Na] |
| 1.55E-06 | 571.2892 |  |  |  |  |
| 2.20E-04 | 571.6282 |  |  |  |  |
| 6.10E-03 | 572.1420 |  |  |  |  |
| 5.80E-03 | 574.9688 |  |  |  |  |
| 1.09E-02 | 575.3032 |  |  |  |  |
| 3.18E-03 | 577.2776 |  |  |  |  |
| 1.84E-02 | 581.2243 |  |  |  |  |
| 9.37E-03 | 584.0837 |  |  |  |  |
| 1.11E-05 | 595.6603 |  |  |  |  |
| 1.00E-06 | 595.9955 |  |  |  |  |
| 1.27E-03 | 603.2107 |  |  |  |  |
| 5.04E-05 | 612.3242 |  |  |  |  |
| 6.08E-07 | 612.6609 |  |  |  |  |
| 3.23E-04 | 622.2159 |  |  |  |  |
| 1.56E-02 | 641.5171 | **89.4** | DG(18:3(9Z,12Z,15Z)/20:3(8Z,11Z,14Z)/0:0)[iso2] | C41H68O5 | [M+H] |
| 1.56E-02 | 641.5171 | **89.4** | DG(18:1(9Z)/18:2(9Z,12Z)/0:0)[iso2] | C39H70O5 | [M+Na] |
| 1.06E-05 | 655.0230 |  |  |  |  |
| 1.92E-07 | 655.3567 |  |  |  |  |
| 1.09E-07 | 655.6916 |  |  |  |  |
| 9.75E-03 | 666.6613 |  |  |  |  |
| 3.72E-04 | 697.7203 |  |  |  |  |
| 1.63E-02 | 698.3857 |  |  |  |  |
