# Supplementary material for: Detailed Mitochondrial Phenotyping by High Resolution Metabolomics
Source: PLoS One. 2012 Mar 6;7(3):e33020. doi: 10.1371/journal.pone.0033020 (PMC3295783; doi:10.1371/journal.pone.0033020)
Supplement: Table S2 — Annotated mitochondrial metabolites from AE (A) and C18 (B) found to be significant between WT and TG via OPLS-DA. Features found to be significant were searched against the metabolomics databases (MMCD and Metlin) to assign reasonable matches. Metabolites that matched to known human drugs and drug metabolites were excluded. (DOC) [file pone.0033020.s002.doc]

**Table S2.**

A.

| **m/z** | **RT** | **Identity** | **Formula** | **Adduct** |
| --- | --- | --- | --- | --- |
| **134.0443** | **167.8** | Aspartate | C4H7NO4 | [M+H] |
| **165.0749** | **144.1** | 2-Deoxy-glucose | C6H12O5 | [M+H] |
| **168.0686** | **75.6** | Hydroxyphenyl glycine; Pyridoxal (vitamin B6) | C8H9NO3 | [M+H] |
| **183.0855** | **192.6** | Galactitol, Mannitol | C6H14O6 | [M+H] |
| **189.1238** | **67.3** | Glycyl-Leucine | C8H16N2O3 | [M+H] |
|  |  | 2,5-undecadienal | C11H18O | [M+Na] |
| **203.1395** | **66.5** | Dipeptide: Leu/Ile-Ala | C9H18N2O3 | [M+H] |
|  |  | Carnitine | C7H15NO3 | [M+ACN+H] |
|  |  | 3,6,8-dodecatrien-1-ol | C12H20O | [M+Na] |
| **204.9577** | **60.6** |  |  |  |
| **205.0676** | **129.0** | Mannitol | C6H14O6 | [M+Na] |
| **206.0713** | **75.5** | lipoamide | C8H15NOS2 | [M+H] |
| **212.0579** | **167.8** | Topaquinone | C9H9NO5 | [M+H] |
| **219.1345** | **68.0** | Lysopine; Dipeptide: Leu/Ile-Ser, Val-Thr | C9H18N2O4 | [M+H] |
| **221.0414** | **113.1** | Vanillylmandelic acid | C9H10O5 | [M+Na] |
| **233.1501** | **66.8** | Dipeptide - Leu/Ile-Thr | C10H20N2O4 | [M+H] |
|  |  | 3E,5E-tridecadienoic acid | C13H22O2 | [M+Na] |
| **239.1052** | **159.9** | 1-Piperazineethanesulfonic acid, 4-(2-hydroxyethyl)-; Dipeptide: Gly-Tyr | C8H18N2O4S | [M+H] |
|  |  | 10E,12E-tetradecadiene-4,6-diynoic acid | C14H16O2 | [M+Na] |
| **240.1090** | **75.6** | 6-Lactoyl-5,6,7,8-tetrahydropterin | C9H13N5O3 | [M+H] |
| **246.1453** | **69.9** | Dipeptide: Gln-Val, Asn-Leu/Ile; Tripetide: GLG, VGA | C10H19N3O4 | [M+H] |
|  |  | Dipeptide: Val-Ser | C8H16N2O4 | [M+ACN+H] |
| **258.1085** | **106.7** | 5-Methylcytidine, Glycerophosphocholine |  | [M+H] |
| **260.0951** | **76.8** |  |  |  |
| **260.1610** | **67.8** | Dipeptide: Gln-Leu/Ile; Tripeptide: AGL/I, AVA | C11H21N3O4 | [M+H] |
|  |  | Dipeptide: Leu/Ile-Ser, Thr-Val | C9H18N2O4 | [M+ACN+H] |
| **261.0955** | **121.2** |  |  |  |
| **267.0971** | **443.6** | p-aminobenzoyl glutamate | C12H14N2O5 | [M+H] |
|  |  | Dipeptide: Pro-Glu | C10H16N2O5 | [M+Na] |
| **268.1378** | **168.4** | Dipeptide: Arg-Ala | C9H19N5O3 | [M+Na] |
|  |  | Carnosine; Dipeptide: Ala-His | C9H14N4O3 | [M+ACN+H] |
| **277.1018** | **342.3** | Dipeptide: Glu-Glu | C10H16N2O7 | [M+H] |
| **279.1708** | **66.8** | Dipeptide: Leu/Ile-Phe | C15H22N2O3 | [M+H] |
| **280.0918** | **74.1** | Tripeptide: TCG, SCA; Thiamine Acetic Acid | C9H17N3O5S1; C12H15N4O2S | [M+H] |
|  |  | 5-Methylcytidine, Glycerophosphocholine | C10H15N3O5; C8H20NO6P | [M+Na] |
| **282.0767** | **171.7** | 7-(Acetyloxy)-3-(3-pyridinyl)-2H-1-benzopyran-2-one | C16H11NO4 | [M+H] |
| **283.0817** | **76.8** |  |  |  |
| **284.0858** | **78.9** | Cucumopine |  | [M+H] |
|  |  | Dipeptide: Asp-Gln, Asp-Glu; Tripeptide: DAG, GGE | C9H15N3O6 | [M+Na] |
| **295.1661** | **67.4** | Dipeptide: Leu/Ile-Tyr | C15H22N2O4 | [M+H] |
|  |  | Estradiol | C18H24O2 | [M+Na] |
| **299.1261** | **77.5** | Dibenzylsuccinate; 7-methylguanosine | C18H18O4; C11H16N5O5 | [M+H] |
| **300.2878** | **145.8** | Sphingosine | C18H37NO2 | [M+H] |
| **307.0816** | **519.3** | Leucocyanidin | C15H14O7 | [M+H] |
| **314.1066** | **169.2** |  |  |  |
| **316.1222** | **167.4** |  |  |  |
| **320.1263** | **203.1** | Dipeptide: Asp-Trp; Tripeptide: CTP |  | [M+H} |
|  |  | Dipeptide: Glu-Met | C10H18N2O5S1 | [M+ACN+H] |
| **321.0832** | **159.2** |  |  |  |
| **322.1050** | **175.0** | S-Methyl GSH;S-Methylglutathione; gamma-L-Glutamyl-L-cysteinyl-beta-alanine | C11H19N3O6S | [M+H] |
| **323.1431** | **546.1** | 1-Methyladenosine | C11H15N5O4 | [M+ACN+H] |
| **324.0575** | **298.5** | CMP | C9H14N3O8P | [M+H] |
| **327.1573** | **77.7** | Tripeptide: PPN | C14H22N4O5 | [M+H] |
| **329.0695** | **155.5** |  |  |  |
| **332.1255** | **89.9** |  |  |  |
| **339.1014** | **76.4** |  |  |  |
| **340.9332** | **59.9** |  |  |  |
| **341.1718** | **78.1** | Bisphenol A diglycidyl ether, Tripeptide: QPP | C21H24O4; C15H24O5 | [M+H] |
|  |  | Ubiquinone (Q2) | C19H26O4 | [M+Na] |
| **357.1291** | **105.7** | Tripeptide: RCG | C11H22N6O4S1 | [M+Na] |
| **358.1317** | **123.4** | Tripeptide: HDS | C13H19N5O7 | [M+H] |
| **359.1276** | **94.6** |  |  |  |
| **373.1152** | **157.7** | Tripeptide: SMN, QTC | C12H22N4O6S1 | [M+Na] |
| **347.2169** |  | Corticosterone | C21H30O4 | [M+H] |
| **386.6112** | **143.2** |  |  |  |
| **391.1530** | **74.7** | Tripeptide: QDE, NQQ, ADW, | C14H22N4O9; C18H22N4O6 | [M+H] |
| **394.5969** | **135.1** |  |  |  |
| **398.1264** | **525.6** | Tripeptide: CFE | C17H23N3O6S1 | [M+H] |
|  |  | Tripeptide: NQD | C13H21N5O8 | [M+Na] |
| **400.2190** | **67.9** | Tripeptide: RQP | C16H29N7O5 | [M+H] |
|  |  | Tripeptide: KPD | C15H26N4O6 | [M+ACN+H] |
|  |  | Tripeptide: L/IFV | C20H31N3O4 | [M+Na] |
| **406.1439** | **553.0** | Tripeptide: EEE, FCH | C15H23N3O10; C18H23N5O4S1 | [M+H] |
|  |  | Tripeptide: MYA, MSF, CVY, NNH | C17H25N3O5S1; C14H21N7O6 | [M+Na] |
| **407.2289** | **68.6** | Tripeptide: PYK, QFL/I | C20H30N4O5 | [M+H] |
|  |  | Tripeptide: L/IFS, L/IYA | C18H27N3O5 | [M+ACN+H] |
| **408.9206** | **60.5** |  |  |  |
| **412.1935** | **168.3** | Tripeptide: MYV, HQQ | C19H29N3O5S1; C16H25N7O6 | [M+H] |
|  |  | Tripeptide: TNH, QHS | C14H22N6O6 | [M+ACN+H] |
|  |  | Tripeptide: RNT, SRQ, KGW | C14H27N7O6; C19H27N5O4 | [M+Na] |
| **414.1371** | **96.8** | Tripeptide: ETC | C17H23N3O7S1 | [M+H] |
| **415.1407** | **90.6** | Tripeptide: CDR, GMW | C18H24N4O4S1 | [M+Na] |
|  |  | Tripeptide: CHD | C13H19N5O6S1 | [M+ACN+H] |
| **416.1371** | **157.6** | Tripeptide CMY | C15H22N5O7P; C17H25N3O5S2 | [M+H] |
| **417.0703** | **157.4** |  |  |  |
| **426.1661** | **457.7** | Tripeptide: QMF; Hyaluronic acid | C19H27N3O6S1; C16H27NO12 |  |
| **427.0915** | **167.4** | Cysteineglutathione disulfide | C13H22N4O8S2 | [M+H] |
| **428.0960** | **169.3** |  |  |  |
| **429.0950** | **147.7** |  |  |  |
| **430.0914** | **166.1** |  |  |  |
| **431.0947** | **166.9** |  |  |  |
| **433.0432** | **151.3** |  |  |  |
| **438.1215** | **91.9** |  |  |  |
| **446.1773** | **169.6** | Tetrahydrofolate | C19H23N7O6 | [M+H] |
| **449.1812** | **76.2** |  |  |  |
| **453.1324** | **166.1** |  |  |  |
| **453.6347** | **167.2** |  |  |  |
| **454.1359** | **168.8** |  |  |  |
| **461.1191** | **164.2** |  |  |  |
| **461.6203** | **161.1** |  |  |  |
| **462.1211** | **160.1** |  |  |  |
| **467.1803** | **81.2** | Tripeptide: MMT, TDF | C21H30N4O4S2; C24H26N4O6 | [M+H] |
|  |  | Tripeptide: YED | C18H23N3O9 | [M+ACN+H] |
| **470.1582** | **76.5** |  |  |  |
| **471.1641** | **76.0** | Tripeptide: TYC | C23H26N4O5S1 | [M+H] |
| **472.1548** | **309.5** | 10-Formyldihydrofolate | C20H21N7O7 | [M+H] |
| **476.1580** | **167.2** |  |  |  |
| **476.9085** | **60.6** |  |  |  |
| **477.1611** | **167.8** | Tripeptide: FCW | C23H26N4O4S1 | [M+Na] |
| **478.1622** | **167.5** |  |  |  |
| **479.1653** | **166.9** | Tripeptide: HDW | C21H24N6O6 | [M+Na] |
| **479.1620** |  |  |  |  |
| **486.1343** | **75.7** |  |  |  |
| **498.2862** | **54.6** | PS(16:0/0:0) | C22H44NO9P | [M+H] |
|  |  | PG(14:0/0:0)[U] | C20H41O9P | [M+ACN+H] |
| **498.6204** | **54.7** |  |  |  |
| **514.3349** | **69.2** |  |  |  |
| **520.3364** | **478.6** | 1-Linoleoylglycerophosphocholine | C26H50NO7P | [M+H] |
| **521.3385** | **479.1** | PC(18:2(2E,4E)/0:0) | C26H51NO7P | [M+H] |
|  |  | PE(18:1(9Z)/0:0) | C23H46NO7P | [M+ACN+H] |
| **544.3365** | **486.9** | PC(20:4(5Z,8Z,11Z,14Z)/0:0)[U] | C28H50NO7P | [M+H] |
|  |  | PC(O-16:1(11Z)/2:0) | C26H52NO7P | [M+Na] |
| **544.8968** | **60.9** |  |  |  |
| **545.3402** | **483.7** | PC(20:4(5Z,8Z,11Z,14Z)/0:0) | C28H51NO7P | [M+H] |
|  |  | LysoPE(0:0/20:3(11Z,14Z,17Z)) | C25H46NO7P | [M+ACN+H] |
|  |  | PC(P-16:0/2:0) | C26H53NO7P | [M+Na] |
| **548.1912** | **73.0** |  |  |  |
| **568.3349** | **504.6** | PS(10:0/10:0) | C26H50NO10P | [M+H] |
|  |  | LysoPC(20:3(5Z,8Z,11Z)) | C28H52NO7P | [M+Na] |
| **569.3381** | **501.0** | PC(22:6(4E,7E,10E,13E,16E,19E)/0:0)[U] | C30H51NO7P | [M+H] |
|  |  | LysoPE(0:0/22:5(4Z,7Z,10Z,13Z,16Z)) | C27H46NO7P | [M+ACN+H] |
| **581.2211** | **137.6** |  |  |  |
| **603.2018** | **74.8** |  |  |  |
| **604.2052** | **75.2** |  |  |  |
| **612.8828** | **61.4** |  |  |  |
| **622.2053** | **77.0** |  |  |  |
| **680.8716** | **61.4** |  |  |  |
| **696.8464** | **62.5** |  |  |  |

**B.**

| **m/z** | **RT** | **Identity** | **Formula** | **Adduct** |
| --- | --- | --- | --- | --- |
| **184.0730** | **525.3** | Phosphorylcholine | C5H14NO4P | [M+H] |
| **205.0686** | **105.4** |  |  |  |
| **206.0722** | **100.0** |  |  |  |
| **212.0578** | **106.9** |  |  |  |
| **221.0424** | **98.9** |  |  |  |
| **239.1054** | **112.2** |  |  |  |
| **258.1082** | **104.9** | 5-Methylcytidine, glycerophosphocholine | C10H15N3O5 | [M+H] |
| **261.0903** | **102.3** |  |  |  |
| **261.0987** | **134.0** |  |  |  |
| **266.1211** | **112.3** | Thiamine | C12H17N4OS | [M+H] |
| **277.0638** | **96.8** |  |  |  |
| **279.1692** | **106.8** | Dipeptide: Leu/Ile-Phe | C15H22N2O3 | [M+H] |
| **280.0944** | **98.7** | Tripeptide: TCG; CSA |  | [M+H] |
|  |  | Glycerophosphocholine | C8H20NO6P | [M+Na] |
| **281.1376** | **506.6** | 7,8-dihydro-L-Biopterin | C9H13N5O3 | [M+ACN+H] |
| **283.0816** | **104.9** |  |  |  |
| **299.1252** | **103.9** | 7-methylguanosine | C11H16N5O5 | [M+H] |
| **299.1972** | **491.9** |  |  |  |
| **302.3043** | **452.1** | Sphinganine | C18H39NO2 | [M+H] |
| **321.0835** | **125.0** |  |  |  |
| **327.1561** | **103.5** |  |  |  |
| **332.1244** | **105.8** | Dipeptide: Gln-Tyr; Tripeptide: FSG, AGY | C14H19N3O5 | [M+Na] |
| **333.1277** | **105.2** |  |  |  |
| **335.1419** | **120.3** |  |  |  |
| **337.2728** | **508.5** | 2alpha-(Hydroxymethyl)-17-methyl-5alpha-androstane-3beta,17beta-diol | C21H36O3 | [M+H] |
| **339.1010** | **105.9** |  |  |  |
| **341.1713** | **102.4** | Ubiquinone | C19H26O4 | [M+Na] |
| **346.1155** | **105.0** | Tripeptide: HCS | C12H19N5O5S1 | [M+H] |
| **359.1279** | **103.6** |  |  |  |
| **373.1153** | **127.4** | Tripeptide: SMN, CQT | C12H22N4O6S1 | [M+Na] |
| **373.9658** | **89.2** |  |  |  |
| **374.2167** | **90.3** | Tripeptide: ARQ | C14H27N7O5 | [M+H] |
|  |  | Dipeptide: Lys-Trp; Tripeptide: ASR, GTR | C17H24N4O3; C12H24N6O5 | [M+ACN+H] |
| **381.1469** | **113.8** |  |  |  |
| **382.1507** | **126.1** | Tripeptide: FGH | C17H21N5O4 | [M+Na] |
| **385.2723** | **505.8** |  |  |  |
| **386.2757** | **505.2** | Tripeptide: KVV | C16H32N4O4 | [M+ACN+H] |
| **386.6116** | **126.5** |  |  |  |
| **387.1460** | **107.9** | Tripeptide: HCQ | C14H22N6O5S1 | [M+H] |
|  |  | Tripeptide: HCS | C12H19N5O5S1 | [M+ACN+H] |
| **388.1557** | **102.0** |  |  |  |
| **391.1530** | **98.6** |  |  |  |
| **398.1935** | **101.9** |  |  |  |
| **401.7366** | **491.4** |  |  |  |
| **408.2907** | **484.3** |  |  |  |
| **412.2095** | **102.4** | PE(6:0/6:0) | C17H34NO8P | [M+H] |
| **415.1412** | **100.3** | Tripeptide: GMW | C18H24N4O4S1 | [M+Na] |
|  |  | Tripeptide: CHD | C13H19N5O6S1 | [M+ACN+H] |
| **416.1361** | **99.7** |  |  |  |
| **417.1435** | **99.7** |  |  |  |
| **426.3189** | **337.7** |  |  |  |
| **428.0947** | **119.7** |  |  |  |
| **428.2040** | **102.5** | Tripeptide: L/IHH, RKC | C18H27N7O4; C15H31N7O4S1 | [M+Na] |
|  |  | Tripeptide: CHK | C15H26N6O4S1 | [M+ACN+H] |
| **430.1193** | **102.4** |  |  |  |
| **432.1691** | **110.2** | Tripeptide: QDE | C14H22N4O9 | [M+ACN+H] |
| **434.0590** | **223.0** |  |  |  |
| **453.1335** | **168.2** |  |  |  |
| **453.6350** | **150.4** |  |  |  |
| **454.1362** | **135.8** | 1-16:0-lysoPE | C21H44NO7P | [M+H] |
|  |  | Tripeptide: CYF | C21H25N3O5S1 | [M+Na] |
| **454.2916** | **538.5** | PE(16:0/0:0) | C21H44NO7P | [M+H] |
| **461.1191** | **136.6** |  |  |  |
| **461.6208** | **131.5** |  |  |  |
| **462.1794** | **102.1** |  |  |  |
| **467.1783** | **108.2** | Tripeptide: MWM | C21H30N4O4S2 | [M+H] |
|  |  | Tripeptide: YQD | C18H23N3O9 | [M+ACN+H] |
| **468.3426** | **505.5** | PE(O-18:0/0:0) | C23H50NO6P | [M+H] |
| **470.1600** | **102.3** | Tripeptide: TDQ, EYH, ETN | C20H25N5O7 | [M+Na] |
| **471.1644** | **103.7** |  |  |  |
| **474.2157** | **112.9** | Tripeptide: PTM | C21H28N4O4S1 | [M+ACN+H] |
| **478.2916** | **507.5** | Tripeptide: FRR | C21H35N9O4 | [M+H] |
|  |  | PA(18:1(9Z)/0:0) | C21H41O7P | [M+ACN+H] |
| **479.2939** | **508.6** | Tripeptide: KKY | C21H35N5O5 | [M+ACN+H] |
| **482.3223** | **496.0** | PE(18:0/0:0) | C23H48NO7P | [M+H] |
| **486.1352** | **104.5** |  |  |  |
| **488.2405** | **102.7** |  |  |  |
| **494.3211** | **504.0** | PC(16:1(9E)/0:0) | C24H48NO7P | [M+H] |
| **498.6213** | **89.7** |  |  |  |
| **498.9555** | **91.0** |  |  |  |
| **500.2736** | **494.3** | LysoPE(0:0/20:5(5Z,8Z,11Z,14Z,17Z)) | C25H42NO7P | [M+H] |
|  |  | Tripeptide: RFH; PA(20:4(5Z,8Z,11Z,14Z)/0:0) | C21H30N8O4; C23H39O7P | [M+ACN+H] |
|  |  | Tripeptide: RFR | C21H35N9O4 | [M+Na] |
| **502.2911** | **507.1** | PE(20:4(5Z,8Z,11Z,14Z)/0:0) | C25H44NO7P | [M+H] |
|  |  | PE(18:1(9Z)/0:0) | C23H46NO7P | [M+Na] |
| **503.2942** | **507.7** |  |  |  |
| **504.3021** | **516.9** | PE(18:0/0:0) | C23H48NO7P | [M+Na] |
| **518.3220** | **496.7** | PC(18:3(9Z,12Z,15Z)/0:0)[U] | C26H48NO7P | [M+H] |
|  |  | LysoPC(16:0) | C24H50NO7P | [M+Na] |
| **520.3375** | **519.9** | 1-Linoleoylglycerophosphocholine | C26H50NO7P | [M+H] |
| **521.3405** | **523.3** |  |  |  |
| **524.2723** | **508.5** | PE(20:4(5Z,8Z,11Z,14Z)/0:0) | C25H44NO7P | [M+Na] |
| **524.3672** | **496.2** | PC(18:0/0:0)[U] | C26H54NO7P | [M+H] |
| **525.2751** | **508.5** |  |  |  |
| **526.2896** | **506.0** | LysoPE(0:0/20:3(11Z,14Z,17Z)) | C25H46NO7P | [M+Na] |
| **527.2992** | **463.9** | Desmosine | C24H40N5O8 | [M+H] |
| **528.3045** | **513.6** | LysoPE(0:0/22:5(4Z,7Z,10Z,13Z,16Z)) | C27H46NO7P | [M+H] |
|  |  | PE(20:4(5Z,8Z,11Z,14Z)/0:0) | C25H44NO7P | [M+ACN+H] |
|  |  | LysoPE(0:0/20:2(11Z,14Z)) | C25H48NO7P | [M+Na] |
| **542.3208** | **509.6** | PC(20:5(5Z,8Z,11Z,14Z,17Z)/0:0) | C28H48NO7P | [M+H] |
|  |  | 1-Linoleoylglycerophosphocholine | C26H50NO7P | [M+Na] |
| **543.3236** | **510.1** |  |  |  |
| **544.3371** | **517.2** | PC(20:4(5Z,8Z,11Z,14Z)/0:0)[U] | C28H50NO7P | [M+H] |
|  |  | PC(O-16:1(11Z)/2:0) | C26H52NO7P | [M+Na] |
| **545.3418** | **521.5** | PC(P-16:0/2:0) | C26H53NO7P | [M+Na] |
| **546.3008** | **91.5** |  |  |  |
| **546.3448** | **522.5** |  |  |  |
| **547.1835** | **169.0** |  |  |  |
| **548.1946** | **99.5** |  |  |  |
| **548.2729** | **505.9** |  |  |  |
| **549.1968** | **100.2** |  |  |  |
| **549.2764** | **506.4** |  |  |  |
| **558.2934** | **525.3** |  |  |  |
| **564.2467** | **506.7** |  |  |  |
| **566.3196** | **522.7** | PC(0:0/20:4(5Z,8Z,11Z,14Z)) | C28H50NO7P | [M+Na] |
| **567.3230** | **528.9** |  |  |  |
| **568.3366** | **518.6** | LysoPC(22:6(4Z,7Z,10Z,13Z,16Z,19Z)) | C30H50NO7P | [M+H] |
|  |  | LysoPC(20:3(5Z,8Z,11Z)) | C28H52NO7P | [M+Na] |
| **569.1622** | **179.4** |  |  |  |
| **569.3398** | **518.2** |  |  |  |
| **570.3447** | **518.1** |  |  |  |
| **577.2776** | **445.2** |  |  |  |
| **590.3186** | **518.4** | LysoPC(22:6(4Z,7Z,10Z,13Z,16Z,19Z)) | C30H50NO7P | [M+Na] |
| **591.3230** | **518.9** | D-Urobilinogen | C33H42N4O6 | [M+H] |
| **603.2107** | **100.8** | 10-Formyltetrahydrofolyl L-glutamate | C25H30N8O10 | [M+H] |
| **604.2148** | **102.1** |  |  |  |
| **606.2929** | **519.3** |  |  |  |
| **622.2159** | **97.5** |  |  |  |
| **623.4993** | **527.8** |  |  |  |
| **624.5038** | **525.4** |  |  |  |
| **641.1149** | **90.3** |  |  |  |
| **641.3173** | **88.9** |  |  |  |
| **641.5171** | **89.4** | DG(18:2(9Z,12Z)/20:4(5Z,8Z,11Z,14Z)/0:0)[iso2] | C41H68O5 | [M+H] |
| **740.5188** | **527.8** | 18:1-18:3-PE | C41H74NO8P | [M+H] |
|  |  | PC(16:0/15:1(14)) | C39H76NO8P | [M+Na] |
| **741.5213** | **527.2** | PC(16:0/15:1(14)) | C39H77NO8P | [M+Na] |
|  |  | PE(15:0/18:3(6Z,9Z,12Z)) | C38H70NO8P | [M+ACN+H] |
| **758.5659** | **524.4** | 18:1-18:1-PE | C42H80NO8P | [M+H] |
|  |  | DG(22:5(7Z,10Z,13Z,16Z,19Z)/22:5(7Z,10Z,13Z,16Z,19Z)/0:0) | C47H72O5 | [M+ACN+H] |
| **759.5677** | **522.8** | PC(16:0/15:1(14)) | C39H76NO8P | [M+ACN+H] |
| **760.5778** | **523.0** | PC(14:0/20:1(11Z)) | C42H82NO8P | [M+H] |
|  |  | PC(16:0/15:1(14)) | C39H77NO8P | [M+ACN+H] |
| **761.5845** | **521.5** | PE(17:0/17:0)[U] | C39H78NO8P | [M+ACN+H] |
| **764.5157** | **528.6** | 20:3-18:3-PE | C43H74NO8P | [M+H] |
|  |  | PE(18:0/18:3(9Z,12Z,15Z))[U] | C41H76NO8P | [M+Na] |
| **765.5212** | **527.4** | PC(14:1(9Z)/18:4(6Z,9Z,12Z,15Z)) | C40H70NO8P | [M+ACN+H] |
| **766.5277** | **532.0** |  |  |  |
| **767.5326** | **529.1** | PC(14:0/18:4(6Z,9Z,12Z,15Z)) | C40H72NO8P | [M+ACN+H] |
| **782.5655** | **523.3** | PC(16:0/20:4(5Z,8Z,11Z,14Z)) | C44H80NO8P | [M+H] |
|  |  | PC(14:0/20:1(11Z)) | C42H82NO8P | [M+Na] |
| **784.5818** | **525.6** | PC(14:1(9Z)/22:2(13Z,16Z)) | C44H82NO8P | [M+H] |
|  |  | PE(17:0/20:0) | C42H84NO8P | [M+Na] |
| **785.5830** | **523.9** | PC(10:0/24:0) | C42H85NO8P | [M+Na] |
|  |  | PE(18:1(9Z)/18:1(9Z))[U] | C41H78NO8P | [M+ACN+H] |
| **790.5321** | **528.0** | PE(18:1(11Z)/22:6(4Z,7Z,10Z,13Z,16Z,19Z)) | C45H76NO8P | [M+H] |
|  |  | PA(18:0/22:6(4Z,7Z,10Z,13Z,16Z,19Z)) | C43H73O8P | [M+ACN+H] |
|  |  | PE(18:0/20:4(5Z,8Z,11Z,14Z))[U] | C43H78NO8P | [M+Na] |
| **791.5385** | **529.0** | PC(15:0/20:4(5Z,8Z,11Z,14Z))[U] | C43H79NO8P | [M+Na] |
|  |  | PC(14:1(9Z)/20:5(5Z,8Z,11Z,14Z,17Z)) | C42H72NO8P | [M+ACN+H] |
| **792.5498** | **527.0** | GPEtn(18:0/22:6(4Z,7Z,10Z,13Z,16Z,19Z)) | C45H78NO8P | [M+H] |
|  |  | PC(15:0/20:3(5Z,8Z,11Z)) | C43H80NO8P | [M+Na] |
| **810.5970** | **525.7** | 1-Stearoyl-2-Arachidonoyl PC | C46H84NO8P | [M+H] |
|  |  | PC(14:0/22:1(13Z)) | C44H86NO8P | [M+Na] |
| **811.6007** | **524.5** | 20:2-18:2-PC | C46H85NO8P+ | [M+H] |
|  |  | PC(15:0/20:3(5Z,8Z,11Z)) | C43H80NO8P | [M+ACN+H] |
|  |  | PC(16:0/20:1(11Z))[U] | C44H87NO8P | [M+Na] |
| **834.5932** | **526.3** | PE(21:0/22:6(4Z,7Z,10Z,13Z,16Z,19Z)) | C48H84NO8P | [M+H] |
|  |  | PC(16:1(9Z)/22:2(13Z,16Z)) | C46H86NO8P | [M+Na] |
